# A Uniquely Complex Mitochondrial Proteome from *Euglena gracilis*

**DOI:** 10.1093/molbev/msaa061

**Published:** 2020-04-05

**Authors:** Michael J Hammond, Anna Nenarokova, Anzhelika Butenko, Martin Zoltner, Eva Lacová Dobáková, Mark C Field, Julius Lukeš

**Affiliations:** m1 Biology Centre, Institute of Parasitology, Czech Academy of Sciences, České Budějovice, Budweis, Czech Republic; m2 Faculty of Sciences, University of South Bohemia, České Budějovice, Budweis, Czech Republic; m3 Faculty of Science, University of Ostrava, Ostrava, Czech Republic; m4 School of Life Sciences, University of Dundee, Dundee, United Kingdom; m5 Faculty of Science, Charles University, Biocev, Vestec, Czech Republic

**Keywords:** mitochondria, proteome, protist, Euglenozoa, *Euglena gracilis*

## Abstract

*Euglena gracilis* is a metabolically flexible, photosynthetic, and adaptable free-living protist of considerable environmental importance and biotechnological value. By label-free liquid chromatography tandem mass spectrometry, a total of 1,786 proteins were identified from the *E. gracilis* purified mitochondria, representing one of the largest mitochondrial proteomes so far described. Despite this apparent complexity, protein machinery responsible for the extensive RNA editing, splicing, and processing in the sister clades diplonemids and kinetoplastids is absent. This strongly suggests that the complex mechanisms of mitochondrial gene expression in diplonemids and kinetoplastids occurred late in euglenozoan evolution, arising independently. By contrast, the alternative oxidase pathway and numerous ribosomal subunits presumed to be specific for parasitic trypanosomes are present in *E. gracilis*. We investigated the evolution of unexplored protein families, including import complexes, cristae formation proteins, and translation termination factors, as well as canonical and unique metabolic pathways. We additionally compare this mitoproteome with the transcriptome of *Eutreptiella gymnastica*, illuminating conserved features of Euglenida mitochondria as well as those exclusive to *E. gracilis*. This is the first mitochondrial proteome of a free-living protist from the Excavata and one of few available for protists as a whole. This study alters our views of the evolution of the mitochondrion and indicates early emergence of complexity within euglenozoan mitochondria, independent of parasitism.

## Introduction

The mitochondrion is an important and versatile organelle with core activities in energy production, iron-sulfur cluster (ISC) biosynthesis, regulation of apoptosis, and metabolism of lipids and amino acids. Arising from the endosymbiosis of an alpha-proteobacterium, before evolving into a truly integrated organelle, mitochondria played a leading role in eukaryogenesis (Lane and Martin 2010). With the remarkable exception of a single known truly amitochondriate protist group (Karnkowska et al. 2016), all extant eukaryotes retain mitochondria or mitochondrion-related organelles which claim descent from the mitochondria of the last eukaryotic common ancestor (Roger et al. 2017).

Although the alpha-proteobacterial ancestor is reconstructed as possessing a genome of ∼5,000 protein-coding genes (Boussau et al. 2004), all but a few genes in modern mitochondria have been either transferred to the nuclear genome or lost. Therefore, most mitochondrial proteins are imported from the cytosol via a specialized translocation apparatus (Mokranjac and Neupert 2009). Accordingly, the mitochondrial genome is of limited use for predicting the total mitochondrial proteome (mitoproteome) and its functions. Indeed, the proteins of modern mitochondria are derived from multiple eukaryotic and prokaryotic sources, with only a small fraction estimated to be contributed by the original endosymbiont (Gray 2015). Although many in silico methods predict localization to mitochondria (Guda et al. 2004; Fukasawa et al. 2015), these predictions are hampered by variability in mitochondrial targeting signals and mechanisms (Santos et al. 2018), as well as divergence across the eukaryotic domain (Emanuelsson et al. 2007; Fukasawa et al. 2017). Thus, direct proteomic approaches remain essential for determining a mitoproteome.

Mitoproteomes have been established for a few unicellular (Sickmann et al. 2003; Casaletti et al. 2017) and multicellular animals, fungi (Taylor et al. 2003; Li et al. 2009; Calvo et al. 2016), and plants (Heazlewood et al. 2003; Hochholdinger et al. 2004; Lee et al. 2013; Mueller et al. 2014). Considering their abundance and diversity, protists remain underinvestigated in this regard, and only mitoproteomes for parasitic *Trypanosoma brucei* (Panigrahi et al. 2009), free-living *Tetrahymena thermophila* (Smith et al. 2007), *Chlamydomonas reinhardtii* (Atteia et al. 2009), and *Acanthamoeba castellanii* (Gawryluk et al. 2014), as well as the mitochondria-related organelles of parasitic *Trichomonas vaginalis* (Schneider et al. 2011), *Giarda intestinalis* (Jedelský et al. 2011), and *Entamoeba histolytica* have been studied in some detail (Mi-ichi et al. 2009).

The protist *Euglena gracilis* arguably represents one of the most comprehensively studied organisms within Euglenida, a group of diverse flagellates distinguished by a striated cell surface or pellicle (Adl et al. 2019). Euglenida belong to the phylum Euglenozoa along with Diplonemea, a group of marine flagellates recently found to consist of high abundance and diversity (Flegontova et al. 2016), and Kinetoplastea, a group notable for numerous parasite members of public health importance (Gibson 2017). From an evolutionary perspective, the extensively studied kinetoplastids are more distant to euglenids than diplonemids (Vesteg et al. 2019). Therefore, any common traits shared between euglenids and kinetoplastids likely represent features possessed by the euglenozoan common ancestor and are expected to be distributed throughout extant members of the phylum. Moreover, their basal phylogenetic position makes euglenids important from an evolutionary perspective, especially since close relatives evolved extremely complex systems for mitochondrial RNA editing and/or *trans*-splicing (Read et al. 2016; Faktorová et al. 2018).

Euglenids are versatile organisms that possess a variety of nutritional strategies, including eukaryotrophy, bacteriotrophy, and osmotrophy (Leander et al. 2017). *Euglena gracilis* can additionally employ photosynthesis due to the presence of a triple membrane-bound plastid acquired through a secondary endosymbiotic event (Zakryś et al. 2017) and thanks to an anaerobically capable mitochondrion, which generates energy via fatty acid fermentation, can grow in anoxic environments (Zimorski et al. 2017). The anaerobically produced wax esters that result from this fermentation are of biotechnological interest as a source of biofuel (Inui et al. 2017), along with a number of other compounds, such as the storage polysaccharide paramylon and essential amino acids which also serve as food supplements in parts of Asia (Krajčovič et al. 2015).

Recent advances in elucidating the molecular biology of *E. gracilis* include the sequencing of a draft genome, transcriptome and determining a whole cell proteome, identifying a large genome in excess of 500 Mb with a comparatively small coding region (<1%) that nonetheless is estimated to include over 36,000 protein-coding genes (Ebenezer et al. 2019). Comparative transcriptomes for cells grown in light versus dark (Ebenezer et al. 2019), rich versus minimal media (O’Neill et al. 2015), and anoxic conditions have been described (Yoshida et al. 2016). Subcellular analyses are also being pursued, with the completion of a chloroplast proteome, revealing a total of 1,345 proteins of multiple origins and a seemingly divergent protein translocation apparatus (Novák Vanclová et al. 2020). The *E. gracilis* mitochondrial genome revealed only seven protein-coding genes and no evidence for posttranscriptional editing and/or splicing that are extensive in sister lineages (Dobáková et al. 2015). In silico prediction of the mitoproteome was estimated at ∼1,100 proteins (Ebenezer et al. 2019), representing a cohort similar to the related *T. brucei* (Peikert et al. 2017), albeit with the caveats discussed above.

Here, we report a mitoproteome for *E. gracilis*, obtained from purified organelles and analyzed by liquid chromatography-mass spectrometry, which contains 1,786 proteins, and is complemented by in silico analysis. Notably, we report the identification of five of seven mitochondrially encoded proteins. We find no evidence for complex RNA editing and processing machineries orthologous with the mitochondrion of kinetoplastids and diplonemids, yet still encountered metabolic and structural complexity that challenges the assumption that protist mitochondria have compositional simplicity.

## Results and Discussion

### Construction of a *Euglena* Mitoproteome

**Fig. 1.**
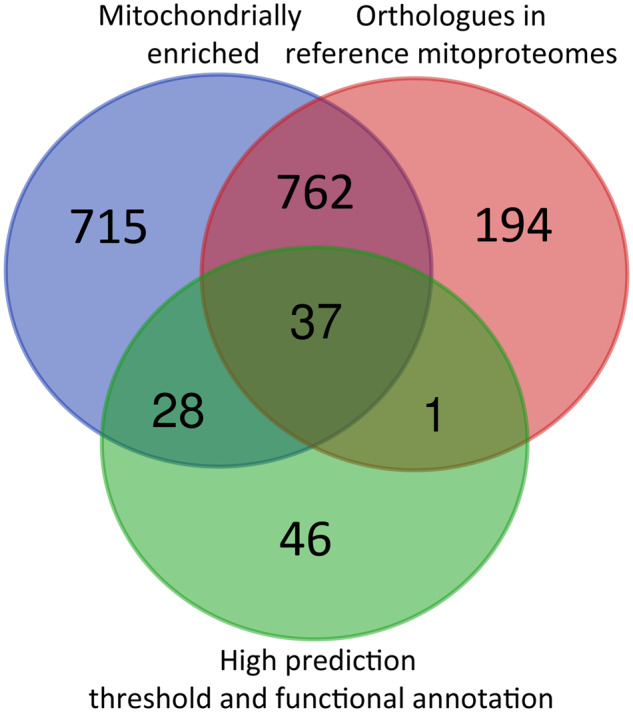
Diagram of identifying strategies used for majority ID transcripts of *Euglena gracilis* mitoproteome. List of proteins grouped by identification strategy is available in supplementary table 4, Supplementary Material online.

For experimental determination of the mitoproteome, pelleted cells were disrupted by sonication, treated with DNaseI, and subjected to discontinuous sucrose density gradient centrifugation, which clearly separated several distinct cellular fractions. The mitochondrial fraction formed a sharp and rather narrow band at the 1.5 and 1.75 M sucrose interface (supplementary fig. 1, Supplementary Material online). This mitochondrial fraction initially yielded 2,704 candidate proteins, of which 994 were orthologous to proteins in reference mitoproteomes, 1,543 were mitochondrially enriched in comparison to both the chloroplast fraction and the whole cell, and 77 had a majority consensus of mitochondrial targeting based on signal peptide predictions (fig. 1).

Together, the verified (i.e., experimentally determined and in silico validated) *E. gracilis* mitoproteome consists of 1,786 majority identified proteins, which were distributed into 1,756 protein groups (supplementary tables 2 and 4, Supplementary Material online). Of these, 4 sequences have been shown to be encoded by the organellar genome (supplementary table 2, Supplementary Material online) and the remaining 1,782 are of nuclear origin (supplementary file 1, Supplementary Material online). This total cohort excludes 24 proteins which were identified through functional annotation as likely contaminants for their clear nonmitochondrial functions but includes another 35 proteins, which were initially excluded based on low enrichment values, but reintegrated based on predicted mitoproteome data and their functional annotation, indicating clear mitochondrial function (supplementary table 4, Supplementary Material online).

Excluding razor and redundant peptides, 1,667 protein groups were identified with more than one unique peptide, whereas 85 were identified from a single unique peptide only (supplementary table 2, Supplementary Material online). The proteome showed wide variation in protein molecular weight, ranging from 416.09 to 10.33 kDa, with 1,307 proteins exhibiting a predicted molecular weight <50 kDa, representing ∼74% of all experimentally verified proteins. When compared with the predicted mitoproteome, 552 of the 1,092 previously predicted proteins were experimentally verified (Ebenezer et al. 2019). Other organisms also show similar differences when confirming the expressed proteome, with two-thirds of the *Arabidopsis thaliana* predicted mitoproteome still to be recovered as expressed proteins (Lee et al. 2013). As mentioned previously, 35 of these predicted proteins were initially rejected due to insufficient mitochondrial enrichment; 12 had greater enrichment in the chloroplast and 23 in whole cells, but these were ultimately included due to their functional annotation and previous transcriptome-based prediction (Ebenezer et al. 2019) (supplementary table 4, Supplementary Material online). Together with unconfirmed in silico sequences (Ebenezer et al. 2019) and the additional sequences identified in this study (supplementary file 2, Supplementary Material online), we currently expect the complete mitoproteome of *E. gracilis* to consist of ∼2,500 proteins (supplementary table 5, Supplementary Material online). Although mitochondrial enrichment represents an obvious criterion for verifying presence within the organelle, there are clearly exceptions to be accounted for, particularly proteins with multiple localizations.

Indeed, of the verified mitoproteome, 211 proteins showed greater enrichment in the chloroplast fraction (supplementary fig. 2, Supplementary Material online), which were retained based on the presence of orthologs in reference mitoproteomes and/or high likelihood of mitochondrial targeting sequence, indicating a very low level of contamination of the chloroplast fraction with mitochondrial components. Importantly, the vast majority (1,567) shows mitochondrial fractional enrichment, with volcano plots revealing minimal contamination with other organelle components (supplementary fig. 3 and table 6, Supplementary Material online), supporting the validity of the mitoproteome predictions. Of the experimentally verified fraction, less than half of proteins (716) were found to possess a mitochondrial import signal in at least one of the available *E. gracilis* transcriptomes as predicted via TargetP, likely arising from variability of import signals and 5′ truncation of some cDNA sequences, highlighting the weakness of relying solely on target prediction software. Bioinformatic predictions of dual-targeted proteins are notoriously difficult, but certain protein families found in the mitochondrial matrix have shown a tendency for plastid presence as well, including those involved in nucleotide metabolism, DNA replication, tRNA biogenesis, and translation (Carrie and Small 2013), which we consider the most likely candidates within our mitoproteome for dual-localization.

**Fig. 2.**
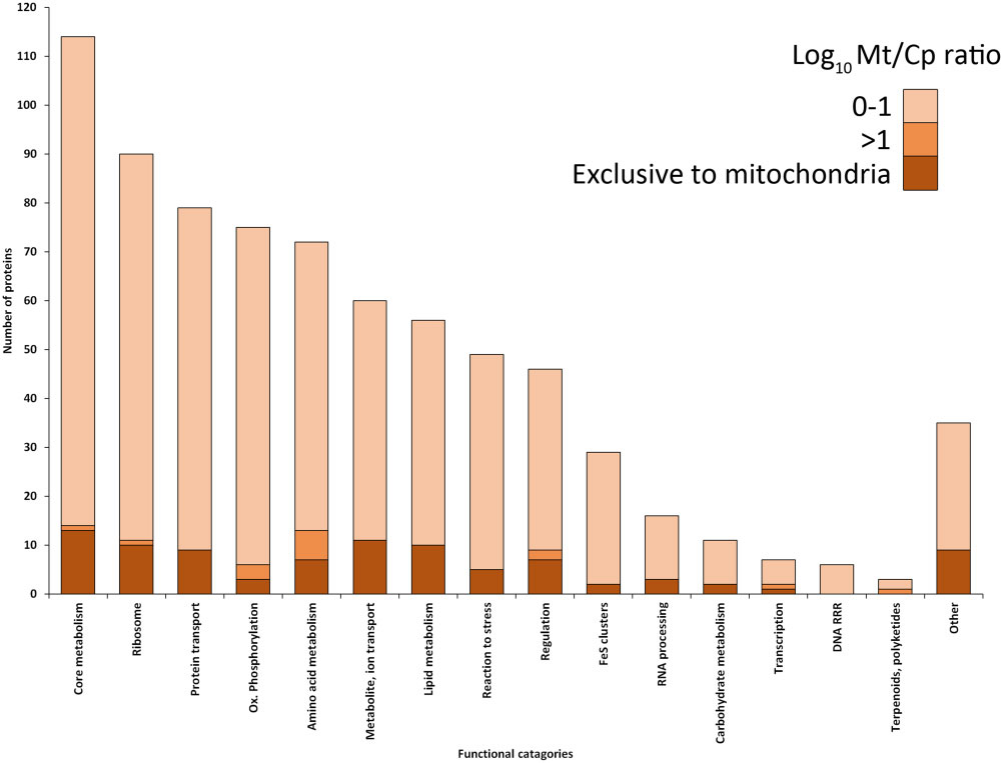
Functional categories of 743 mitochondrially enriched transcripts with predicted function (42% of the entire proteome) including the logarithmic enrichment ratio of the mitochondrial versus chloroplast preparation (log 10MT/CP) ratio for each category, shown in shades of orange (infinite, 0–1 representing 1–10× greater protein amount in mitochondrial fraction, 1–2 for 10–100×; full category names are listed in Materials and Methods).

Functional annotation could be assigned to 788 proteins, leaving an unexpected total of 998 (56%) proteins with unknown function. Of the annotated proteins, “core metabolic pathways,” “ribosome, aminoacyl-tRNA biosynthesis and translation,” and “protein transport, folding, processing and degradation” were most represented (fig. 2). When compared with the categorization of the predicted mitoproteome, we see notable increase in the proportion of proteins with functions attributed to the “ribosome” and “protein transport” categories as well as a reduction in “oxidative phosphorylation and electron transport proteins” (supplementary fig. 4, Supplementary Material online).

### The *E. gracilis* Mitoproteome Is Complex

The *E. gracilis* mitoproteome is larger than that predicted for all other eukaryotes, for which well-curated mitoproteomes based on both predictions and experimental data are available, and specifically *A. thaliana* (843) (Lee et al. 2013), *T. brucei* (1,120) (Peikert et al. 2017), *Saccharomyces cerevisiae* (1,187) (Gonczarowska-Jorge et al. 2017), and *Mus musculus* (1,158) (Calvo et al. 2016). Although varied criteria are often used in different studies to determine genuine mitochondrial components making direct comparisons and estimates of reliability difficult, we consider the number of *E. gracilis* mitoproteins to be notable, especially as the estimated proteome sizes in these other model organisms were initially reported as lower (Werhahn and Braun 2002; Chang et al. 2003; Sickmann et al. 2003; Panigrahi et al. 2009) and have steadily increased over the past 20 years through improved protein identification, increased mass spectrometry sensitivity, and experimental mitochondrial analysis (Calvo et al. 2016; Gonczarowska-Jorge et al. 2017; Peikert et al. 2017).

Moreover, when compared with the high confidence protist mitoproteomes of *Acanthamoeba castellanii* (709) (Gawryluk et al. 2014), *Tetrahymena thermophila* (573) (Smith et al. 2007), and *C. reinhardtii* (347) (Atteia et al. 2009), the complexity of the analyzed mitoproteome is even more impressive. *Euglena gracilis* displays the largest number of unknown sequences (57%) for any mitoproteome surveyed thus far, with corresponding frequencies in other mitoproteomes ranging from 17% in *C. reinhardtii* to 53% in *T. brucei*.

With ∼2,500 sequences, the total predicted mitoproteome of *E. gracilis* (supplementary table 5, Supplementary Material online) represents a size more typical of plant mitochondria, as several software prediction analyses of *A. thaliana* have consistently predicted 2,000–3,000 proteins residing within its mitochondria (Heazlewood et al. 2004; Millar et al. 2005; Lee et al. 2013). We suggest that a common factor contributing to potentially accelerated mitochondrial complexity is the presence of the plastid. *Euglena gracilis* has numerous proteins dually localized to both mitochondria and plastids, and additionally carries some plastid-derived pathways within the mitochondria, as well as what appears to be metabolic cycles which complement plastid function, indicating that plastid interactions with the mitochondrion can foster an overall greater complexity. In contrast to *E. gracilis*, experimental verification of many predicted plant mitoproteomes has remained challenging (Hochholdinger et al. 2004; Huang et al. 2009; Mueller et al. 2014). Indeed, experimentally verified mitoproteomes >1,000 sequences remain a rarity (Salvato et al. 2014), a situation thought to reflect difficulty in detecting hydrophobic and low-abundance proteins (Millar et al. 2005). Recent investigations into the subcellular compartmentalization of metabolic pathways in *E. gracilis* have been lacking extensive proteomic information on the mitochondria (Inwongwan et al. 2019), which we hope to rectify here.

### Mitochondrial Termination Factor Proteins

Mitochondrial termination factor (mTERF)-like proteins are involved in transcription termination/activation and ribosome biogenesis (Roberti et al. 2009). Although initially discovered in mitochondria, mTERFs are also present in chloroplasts, sometimes being dually localized (Kleine and Leister 2015). Whereas four groups of mTERFs have been described in vertebrates, plants have undergone a major expansion with 31 and 35 mTERFs in *Zea mays* and *A. thaliana*, respectively (Kleine and Leister 2015), of which 10 *A. thaliana* mTERFs have been confirmed by proteomics as mitochondrial (Lee et al. 2013). Of an unprecedented 192 mTERFs identified in the *E. gracilis* transcriptome, at least 8 are identified in the mitoproteome (supplementary table 7, Supplementary Material online), of which 4 show exclusive localization to the mitochondrion, raising the possibility that the remaining 4 have dual localizations. We observed six additional mTERFs with considerable enrichment in the chloroplast (supplementary table 7, Supplementary Material online), suggesting that *E. gracilis* also partitions its mTERF family between these two endosymbiont-derived organelles. This raises questions of why *E. gracilis* contains such a large suite of mTERF proteins. The large mitochondrial genomes of plants, which exhibit considerable gene rearrangement and numerous introns, may require greater transcriptional regulation facilitated by the additional mTERFs (Kleine and Leister 2015). However, such an explanation does not seemingly apply to *E. gracilis* which, while containing fragmented mitoprotein-coding genes, nonetheless has a reduced set, importantly lacking the complex posttranscriptional modifications seen in other euglenozoan lineages (Dobáková et al. 2015).

Experimental studies suggest that in plants the expanded mTERF family enables functional diversification, fostering resistance to various abiotic stresses such as increased light, heat, and salt concentrations (Quesada 2016; Robles et al. 2018), whereas mTERF18 confers heat tolerance to *A. thaliana* through regulating redox-related gene expression (Kim et al. 2012). mTERF-like gene MOC1-deficient mutants of *C. reinhardtii* display reduced growth under high light intensity (Schönfeld et al. 2004). Adaptions to abiotic stress may underly the dramatic expansion of mTERFs in *E. gracilis*. Moreover, since in metazoans mTERFs have also been observed mediating ribosome biogenesis (Wredenberg et al. 2013), we suggest that the expanded set of ribosomal subunits in *E. gracilis* (see below) may require additional mTERFs for proper ribosome assembly and/or function.

### RNA Editing and Processing

Kinetoplastids and diplonemids developed highly complex, yet distinct systems of RNA editing and processing of their mitochondrial transcripts. Although in kinetoplastids uridines are extensively inserted into and deleted from mRNAs (Read et al. 2016), in diplonemids mRNAs of systematically fragmented genes undergo *trans*-splicing and C-to-U, A-to-I, and G-to-A, as well as U- and A-appendage editing (Valach et al. 2017; Kaur et al. 2020). Although *E. gracilis* does not have RNA editing (Dobáková et al. 2015), we raise the question of whether the common euglenozoan ancestor possessed preadaptations for these baroque mechanisms.

From the ∼90 proteins involved in mitochondrial RNA editing and processing in *T. brucei*, only homologs to 12 proteins were present in the *E. gracilis* mitoproteome: MRB3010, RBP16, RGG2, PAMC, KPAF1, MHEL61, KRET2, KREX2, poly(A) polymerase, p22 precursor, a PAMC (polyadenylation mediator complex) component Tb927.6.3350, and pentatricopeptide (PPR) protein Tb927.10.10160. An additional 19 proteins are predicted to be involved in mitochondrial RNA processing in *E. gracilis*. These include six DEAD-box RNA helicases, one PPR protein, eight and two proteins similar to MRB3010 and RGG2, respectively, and two splicing factors belonging to the arginine/serine-rich 4/5/6-like family. Remarkably, these splicing factors have homologs only in diplonemids (our unpublished data). Our data indicate that although the last common euglenozoan ancestor did not perform mitochondrial RNA editing, it already had a basic set of RNA processing proteins in its mitochondrion that were repurposed in the evolution of kinetoplastids and potentially diplonemids as key RNA editing enzymes. Those include RNA binding proteins, RNA helicases, terminal uridyl-transferase (TUTase), and the exonuclease listed above. This prompts the question of which preadaptions within the common euglenozoan ancestor triggered emergence of highly complex RNA editing and/or processing systems. One driving force might have been an increasingly scrambled mitochondrial genome (Flegontov et al. 2011). The emergence and evolution of excessive molecular complexity are best explained via purely neutral drift, which becomes irreversible due to rachet mechanisms preventing subsequent simplification (Stoltzfus 1999; Lukeš et al. 2011; McShea and Hordijk 2013).

### Mitochondrial-Encoded Proteins

The mitochondrial genome of *E. gracilis* encodes only seven proteins (nad1, nad4, nad5, cob, cox1, cox2, and cox3), all of which are respiratory complex subunits (Dobáková et al. 2015). Our data revealed nad1 of complex I, cob of complex III, and cox1, 2, and 3 of complex IV, providing proteomic evidence for all complexes consisting of mitochondrial-encoded subunits (supplementary table 2, Supplementary Material online). In trypanosomes, recovery of these complexes has required specific extraction procedures (Horváth et al. 2000; Škodová-Sveráková et al. 2015), and hence detection of five mitochondrially encoded proteins is notable and further demonstrates the quality of our data set. Since the transcriptome was assembled from polyadenylated RNAs (Ebenezer et al. 2019), the presence of cox3 was surprising (supplementary table 4, Supplementary Material online). The status of RNA polyadenylation in the *E. gracilis* mitochondrion is currently unknown (Dobáková et al. 2015), but it is assumed that in most eukaryotes the polyA tails in mitochondrial transcripts are generally shorter than their nuclear-encoded counterparts (Chang and Tong 2012). Although the C-terminal end of cox1 is nuclear-encoded, arising from an ancient gene fission event (Gawryluk and Gray 2010), no sequences showing homology to this region were found in the transcriptome. Cox3-like sequences are also present in the transcriptomes of Yoshida and O’Neill, both of which show a sequence of extended length, whose N-terminal region contains a predicted mitochondrial target peptide. Thus, we suggest cox3 is also dual-encoded, and that the recovered transcript in fact represents the nuclear-encoded region of cox3.

### Respiratory Complexes

So far, 25 conventional respiratory complex subunits have been detected in *E. gracilis* from complex I along with 14 euglenozoan-specific subunits and 20 unknown proteins (Perez et al. 2014; Miranda-Astudillo et al. 2018). Through our analysis, we recovered 19 of these conventional subunits, along with subunit NDUFA8, which has not been identified previously, 7 euglenozoan-specific subunits and 10 unknown proteins. Additionally, we found two potentially novel subunits of complex I (supplementary table 7, Supplementary Material online). It is plausible that some complex I subunits may represent highly diverged paralogs of conventional subunits so far not identified in euglenids, such as Nad2, 3, 4L, and 6, but presumably with potential to increase functional flexibility.

Of complex III, eight conventional and two euglenozoan-specific subunits have been identified, along with three novel proteins (Perez et al. 2014; Miranda-Astudillo et al. 2018). Within this mitoproteome, we recovered five conventional and one euglenozoan-specific subunit, along with all three novel proteins. Cytochrome *c* was detected as three paralogs, indicating duplication events (supplementary table 7, Supplementary Material online). The possibility that *E. gracilis* possesses functional variants of cytochrome *c* is intriguing and recommends itself for further study.

Seven conventional subunits, nine euglenozoan-specific subunits, and eight novel proteins have been identified for complex IV (Perez et al. 2014; Miranda-Astudillo et al. 2018). Nine conventional subunits were also detected in the mitoproteome, including five previously identified ones and four subunits (cox15, cox4, cox11, and cox10) that are new additions. Moreover, we found five euglenozoan-specific subunits and eight previously assigned unknown proteins (supplementary table 7, Supplementary Material online). Finally, although seven conventional and eight euglenozoan-specific subunits have previously been found to encompass the ATP synthase (Perez et al. 2014; Miranda-Astudillo et al. 2018), our mitoproteome contains five subunits of both these groups, as well as two OSCP homologs (supplementary table 7, Supplementary Material online), which again suggests increased functional flexibility.

### Mitochondrial Contact Site and Cristae-Organizing System Complex

The mitochondrial contact site and cristae-organizing system (MICOS) mediates the formation of cristae and increases membrane area available for respiratory complexes (Kozjak-Pavlovic 2017). MICOS supports this function across eukaryotes and may also be involved in protein import into the inner membrane (Kaurov et al. 2018). Although only Mic10 was identified in the *E. gracilis* genome (Ebenezer et al. 2017), we report its proteomic detection along with Mic20, Mic40, Mic60, and a putative Mic34 (supplementary fig. 5, Supplementary Material online). Mic10 typically carries two transmembrane domains, yet these were not predicted for *E. gracilis* with high confidence. The Mic20 of *E. gracilis* contains, same as its ortholog in trypanosomes, a thioredoxin-like domain (supplementary fig. 5, Supplementary Material online), and thus likely also represents a functional analog to Mia40, which is seemingly absent from excavates (Kaurov et al. 2018). Since *T. brucei* Mic60 lacks a mitofilin domain (supplementary fig. 5, Supplementary Material online), which is present in opisthokonts and responsible for interaction with the topogenesis β-barrel (TOB) complex (Kozjak-Pavlovic 2017), it may be unable to fulfill this function (Kaurov et al. 2018). *Euglena gracilis* Mic60 not only lacks a C-terminal mitofilin domain but is also 100 amino acids shorter than its trypanosome ortholog, possessing a transmembrane domain near to the N-terminus. Mic34 of both *E. gracilis* and *T. brucei* carries two coiled-coil domains (supplementary fig. 5, Supplementary Material online), which in the latter has been suggested to support mitofilin-based interactions with the TOB complex (Kaurov et al. 2018). Although Mic34 of *E. gracilis* was initially dismissed because of a high bias HMM score, the presence of two coiled-coil domains (supplementary fig. 5, Supplementary Material online) and detection in the mitoproteome fraction, along with its seemingly essential organellar role, has led us to conclude this as a genuine component. In comparison to the nine MICOS subunits in *T. brucei*, *E. gracilis* presents itself as an evolutionary intermediate between opisthokonts and the expanded and diverged MICOS apparatus of trypanosomes.

### Alternative Respiratory Pathway

In the bloodstream stage of *T. brucei*, an alternative respiratory pathway, composed of glyceraldehyde 3-phosphate dehydrogenase, alternative oxidase (AOX), and a type II alternative NADH dehydrogenase (Verner et al. 2015), is essential. The pathway is present in other life stages in conjunction with the oxidative phosphorylation machinery, where it fosters greater metabolic flexibility in response to nutrient and oxygen availability (Chaudhuri et al. 2006), and also likely plays a role in regulating the level of reactive oxygen species (Fang and Beattie 2003). Both trypanosome-like AOX and glyceraldehyde 3-phosphate dehydrogenase have been identified in *E. gracilis* previously (Perez et al. 2014; Miranda-Astudillo et al. 2018), as well as here (supplementary table 7, Supplementary Material online). For the first time, we also report the detection of type II NADH dehydrogenase. The presence of a full alternative respiratory pathway and multiple paralogs in *E. gracilis* further encourages the view of its mitochondrion as highly versatile and adaptable to a variety of environmental conditions.

### Protein Import: Outer Mitochondrial Membrane

Opisthokonts make use of a translocase of outer membrane (TOM) complex to translocate proteins across the outer mitochondrial membrane (Mani et al. 2016). In kinetoplastids, a diverged atypical translocation of outer membrane (ATOM) complex fulfills the same role (Schneider 2018). Both complexes consist of a central pore-forming subunit, two or more protein receptors, and many smaller subunits (Mani et al. 2016).

**Fig. 3.**
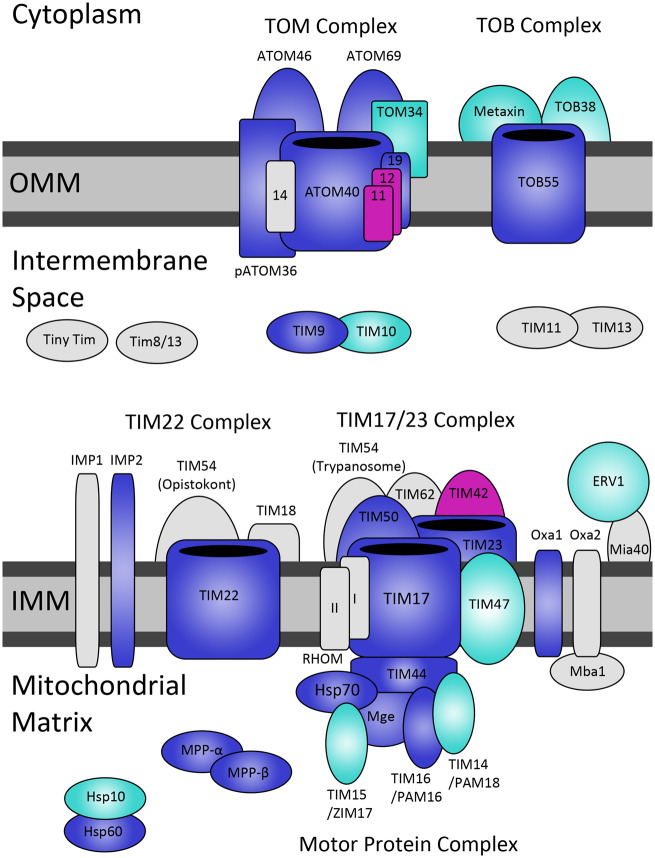
Mitochondrial protein import apparatus of *Euglena gracilis*. Blue for proteomically confirmed sequences, light blue for sequences identified in transcriptome, gray for absent sequences, and purple for “putative” sequences with weak homology that nonetheless show presence within the mitoproteome. Sequences taken from translocation apparatus of *Trypanosoma brucei*, or *Saccharomyces cerevisiae* in the case of opisthokont TIM22 complex and transmembrane proteins, Mba 1, Mia40, and IMP1.

The *E. gracilis* mitoproteome contains 22 proteins associated with import and subsequent processing in the mitochondria that were recovered at the protein level, with an additional 12 identified in silico (supplementary table 7, Supplementary Material online). There are two orthologs of 40 kDa β-barrel pore-forming ATOM (fig. 3), which is essential in related species for proper mitochondrial import through the outer membrane (Eckers et al. 2012). Homologs to ATOM46 and 69 are also present, which in trypanosomes display preference for hydrophobic carrier proteins and presequence containing substrates, respectively (Mani et al. 2016). Another characteristic subunit of the *T. brucei* complex, the peripheral receptor pATOM36, which mediates insertion and assembly of N-terminal anchored outer membrane proteins (Schneider 2018) and was previously assumed to be trypanosome-specific, was also confirmed at protein level (fig. 3). A homolog of TOM34, which in opisthokonts serves as a cochaperone with heat shock proteins for mitochondrial translocation of sequences (Faou and Hoogenraad 2012), was identified in silico (fig. 3). Although a protein similar to ATOM19 was predicted within transcriptome and also recovered in the verified mitoproteome, only weak hits were found for ATOM11 and 12 (fig. 3). Given that opisthokonts also contain four small, stabilizing subunits within their TOM complex (TOM5, 6, 7, and 22, serving a similar role to ATOM11, 12, 14, and 19 in trypanosomatids) (Schneider 2018), it is likely that they are present in *E. gracilis*, but have diverged beyond recognition. Peculiarly, a homolog to ATOM14 and TOM22, which represents the single subunit conserved between opisthokonts and kinetoplastids (Schneider 2018), could not be identified (fig. 3).

TOB55 inserts β-barrel-containing proteins into the outer mitochondrial membrane from the intermembrane space (IMS). In *E. gracilis*, two orthologs of TOB55 were detected by proteomics (supplementary table 7, Supplementary Material online), reminiscent of the duplicated TOB55 of *Trypanosoma cruzi* (Eckers et al. 2012), whereas TOB38 was identified only in silico. TOB55 and TOB38 are essential in fungi (Sharma et al. 2010), whereas the third subunit of the TOB55 complex, SAM37, is dispensable (Desmond et al. 2011). Indeed, this subunit appears absent from *E. gracilis*. Additional peripheral proteins associating with the TOB55 complex with undefined functions are the metaxins, which have been identified in both opisthokonts and trypanosomes (Verner et al. 2015). Two orthologs of metaxin were also identified in the *E. gracilis* transcriptome (supplementary table 7, Supplementary Material online).

### Protein Import: IMS, Inner Mitochondrial Membrane, and Matrix

Translocation of inner membrane (TIM) protein TIM9 of the hexameric TIM9/10 complex was identified at the protein level, whereas only in silico evidence is available for its binding partner TIM10 (fig. 3). The two together traditionally serve to chaperone hydrophobic precursors across the IMS (Wiedemann et al. 2004). We found no orthologs for TIM complex 11/13 or TIM8 and tiny TIMs (fig. 3). The TIM23 complex imports signal-bearing proteins into the inner membrane and the mitochondrial matrix (Wiedemann et al. 2004). We provide mass spectrometric evidence for the existence, in *E. gracilis*, of this complex containing orthologs of TIM16, TIM17, TIM23, TIM44, TIM50, Hsp70, and Mge1 (fig. 3). TIM17, TIM23, and TIM50 constitute the membrane-anchoring component, with TIM50 traditionally working as a receptor for precursors, whereas TIM17 and TIM23 serve as the pore-forming units (Schneider 2018). Moreover, TIM14 and TIM15 were identified in silico (fig. 3) and presumably serve as a part of the motor protein complex and prevent protein aggregation (Fraga et al. 2013).

All trypanosomes studied so far prominently lack the TIM23 pore-forming subunit, presumably forming a channel only via TIM17, suggesting that *E. gracilis* contains a less restricted import apparatus. The presence of two orthologs of TIM17, confirmed by mass spectrometry in *E. gracilis*, is similar to *Trypanosoma cruzi* (Eckers et al. 2012), suggesting that a specific duplication event likely occurred in the common euglenozoan ancestor. Moreover, the absence of TIM21 and Pam17 in both protist species implies that they represent Euglenozoa-specific losses (Verner et al. 2015). *Trypanosoma brucei* exhibits an expanded set of membrane-anchored subunits (TIM47, TIM54, TIM62, TIM42, RHOM 1, and RHOM 2) with undefined functions, of which only TIM47 was identified in our data with confidence (Harsman et al. 2016) (supplementary table 7, Supplementary Material online). A weakly homologous sequence to TIM42 was recovered, which we term a putative protein (supplementary table 7, Supplementary Material online).

The TIM22 complex, which in opisthokonts inserts proteins into the inner mitochondrial membrane (Mokranjac and Neupert 2009), seems to be absent in trypanosomes (Schneider 2018). Hence, the detection by mass spectrometry of an ortholog to the main functional subunit TIM22 (fig. 3) supports the idea of a relatively expanded import system in euglenids, when compared with its highly diverged and streamlined version in trypanosomes. However, other subunits of the TIM22 complex were not found, so that the functional significance remains unclear (fig. 3).

The membrane-anchored Oxa1 is an insertase of the inner membrane (Mokranjac and Neupert 2009), and although an ortholog was identified in the mitoproteome (fig. 3), its binding partner Mba1 was not. Proteins translocated via Oxa1 are associated with removal of IMS sorting signal, undertaken by inner membrane peptidases (IMPs) (Gakh et al. 2002). An IMP2 ortholog is also present but as in *T. brucei* (Verner et al. 2015), IMP1 seems to be absent (fig. 3). In opisthokonts, amoebozoans, and plants, the Erv1-Mia40 complex imports and oxidatively folds small cysteine-rich proteins in the IMS (Allen et al. 2008). Although Erv1 is present in *E. gracilis*, Mia40 could not be identified and is also absent in parasitic chromalveolates (Ebenezer et al. 2019). As proposed for trypanosomes, Mia40 may be functionally complemented by a dedicated subunit of the MICOS complex (Kaurov et al. 2018). *Euglena gracilis* is notable in that it represents the first nonparasitic excavate lacking Mia40, which supports the notion that this protein is absent from euglenozoans (Allen et al. 2008), and may possibly import intermembrane proteins by an as yet unknown pathway.

Proteins transported through the TIM23 complex require removal of the import signal, mediated by the mitochondrial processing peptidase (MPP) complex consisting of α and β subunits. MPP-α recognizes the presequence, whereas MPP-β performs the cleavage (Gakh et al. 2002). As in *T. brucei* (Desy et al. 2012), both proteins were recovered from the mitoproteome (fig. 3). Once cleaved, some proteins will self-assemble, whereas others require assistance, and are folded by heat shock protein (Hsp) 60 and 10 (Martin 1997). Hsp10 was identified in silico, whereas Hsp60 was detected by mass spectrometry (fig. 3).

### Tricarboxylic Acid Cycle

**Fig. 4.**
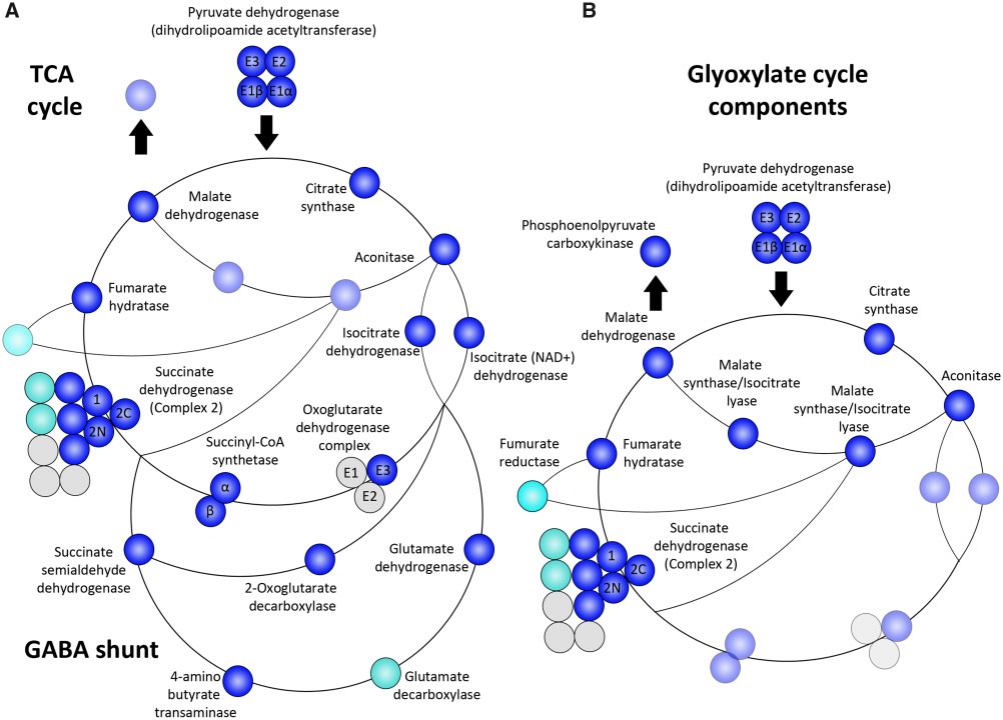
Core metabolic pathways and their proteomic presence within *Euglena gracilis*. (*A*) TCA cycle showing the conventional pathway rendered nonfunctional by absence of key protein subunits along with alpha-proteobacterial shunt and aminobutyric (GABA) shunt. Components involved only in the glyoxylate cycle are transparent. (*B*) Glyoxylate cycle, highlighting associated enzymes from TCA cycle. Blue for proteomically confirmed sequences, light blue for sequences identified in transcriptome, and gray for absent sequences. Duplicated sequences are available in supplementary table 7, Supplementary Material online.

The tricarboxylic acid (TCA) cycle is a key component of all aerobic mitochondria, allowing efficient generation of energy. *Euglena gracilis* employs a modified TCA cycle reminiscent of certain alpha-proteobacteria, where the oxoglutarate dehydrogenase complex is replaced with 2-oxoglutarate decarboxylase and succinate semialdehyde (SSA) dehydrogenase (Green et al. 2000). TCA cycle and associated components show numerous duplication events with multiple copies of key enzymes (supplementary table 7, Supplementary Material online). Under aerobic conditions, pyruvate is transported to the mitochondrion and metabolized by the pyruvate dehydrogenase (PDH) complex, producing acetyl-coenzyme A (CoA), which enters the TCA cycle (Hoffmeister et al. 2004). All four PDH subunits were recovered in vivo (Ebenezer et al. 2019) (fig. 4*A*). The first three steps of the TCA cycle, following the generation of acetyl-CoA, follow a conventional path and all enzymes from these steps (citrate synthase, aconitase, and isocitrate dehydrogenase) were recovered by mass spectrometry (fig. 4*A*). Notably, isocitrate dehydrogenase was among the identified proteins initially rejected based on low mitochondrial enrichment compared with the whole cell but was accepted based on functional annotation.

Traditionally, α-ketoglutarate is catabolized using the α-ketoglutarate dehydrogenase complex, before employing the succinyl-CoA synthetase complex to generate succinate (Zimorski et al. 2017). Again, canonical subunits of succinyl-CoA synthase are present in the mitoproteome, as well as one of three conventional subunits (E3 dihydrolipoyl dehydrogenase) of the α-ketoglutarate dehydrogenase, the other two being absent from the *E. gracilis* genome (Ebenezer et al. 2019), suggesting the pathway is either nonfunctional or uses unconventional components (fig. 4). Retention of both E3 and succinyl-CoA subunits for a seemingly nonfunctional pathway can be explained through their bifunctionality, since E3 exists as a subunit of the PDH complex, while succinyl-CoA synthase also acts in the methylmalonyl-CoA pathway for fatty acid synthesis (fig. 4*A*). *Euglena gracilis* bypasses the need for the α-ketoglutarate dehydrogenase complex through the use of α-ketoglutarate decarboxylase and SSA dehydrogenase. α-Ketoglutarate is first catalyzed to SSA by α-ketoglutarate decarboxylase and then converted to succinate via SSA dehydrogenase (Müller et al. 2012). We find evidence for both essential enzymes of this pathway (fig. 4*A*).

A second variant pathway, the gamma-aminobutyric (GABA) shunt, employs glutamate dehydrogenase, glutamate decarboxylase, and GABA transaminase to generate SSA, which in turn is converted to succinate by SSA dehydrogenase (Zhang et al. 2016). In the mitoproteome, glutamate dehydrogenase and GABA transaminase were detected, whereas glutamate decarboxylase remains identified only in silico (fig. 4*A*). The GABA shunt allows for optimized photosynthetic conditions in cyanobacteria (Nogales et al. 2012), which may also be applicable within *E. gracilis*.

After generation of succinate, the *E. gracilis* TCA cycle proceeds conventionally with succinate dehydrogenase (respiratory complex II) functioning with fumarate hydratase and malate dehydrogenase to complete the cycle, with both of these proteins detected by mass spectrometry. Succinate dehydrogenase is composed of two conserved subunits, one of which, SDH2, is further split into two components (Gawryluk and Gray 2019). These conserved subunits, along with three of eight euglenozoan-specific subunits (SDHTC 6, 7, and 8) were identified (fig. 4*A*). Two genes encoding for euglenozoan-specific subunit 7 were present, likely representing a recent duplication (supplementary table 7, Supplementary Material online).

Notably, *E. gracilis* is one of few organisms to localize the glyoxylate cycle to the mitochondria, as opposed to glyoxysomes (Zimorski et al. 2017). The glyoxylate cycle represents an anabolic variant of the TCA, which enables synthesis of complex carbohydrates from compounds such as lipids, with both cycles sharing several enzymes. In general, the glyoxylate cycle proceeds with the first two TCA reactions, yielding isocitrate, which is then cleaved by isocitrate lyase to form glyoxylate and succinate (Zimorski et al. 2017). Subsequently, glyoxylate is converted to malate via malate synthase, whereas succinate is converted to fumarate by fumarate reductase and further metabolized into malate by fumarate hydratase again. By the activity of malate dehydrogenase, malate is converted into oxaloacetate, which can reenter the TCA cycle, or be processed into phosphoenolpyruvate by phosphoenolpyruvate carboxykinase, initiating gluconeogenesis (Dolan and Welch 2018). To facilitate the glyoxylate cycle, *E. gracilis* uses a bifunctional enzyme, containing domains for both isocitrate lysis and malate synthesis (Nakazawa et al. 2011), which was present within the mitoproteome, whereas fumarate reductase was identified only in silico (fig. 4*B*). Phosphoenolpyruvate carboxykinase was recovered at the protein level, initially rejected for low enrichment, yet eventually recalled (fig. 4*B*). The *E. gracilis* mitochondrion can additionally initiate gluconeogenesis by converting pyruvate directly to oxaloacetate via pyruvate carboxylase, which was proteomically detected (supplementary table 7, Supplementary Material online), and is then processed through the activity of phosphoenolpyruvate carboxykinase. Our analysis of the TCA cycle and associated pathways demonstrates a highly versatile organelle, able to generate and store ATP using a variety of energy sources under a range of conditions; the presence of paralogs for several critical enzymes also reinforces the potential for environmental flexibility.

### Mitochondrial Ribosomes

One hundred and eight mitochondrial ribosomal (mitoribosomal) subunits were identified in silico, representing 68 additional subunits in comparison to the earlier reported complement (Ebenezer et al. 2019). A total of 83 of these were experimentally verified, consisting of 47 large and 34 small mitoribosomal subunits with 2 unclassified (supplementary table 7, Supplementary Material online). From a phylogenetic perspective, these are further classified into a variety of categories: “Core” subunits are of alpha-proteobacterial origin, likely present in the last common eukaryotic ancestor, whereas “accessory” subunits subsequently emerged in different eukaryotic lineages. Thirty-five core and 15 accessory mitoribosomal proteins were recovered, with an additional 6 and 2 in silico predicted but not detected, respectively. Compared with the well-studied mitoribosomes of *T. brucei*, 20 subunits (L5, L14, L29, L30, L33, L35, L41, L2, L48, L53, S6, S14, S21, S26, S30, S33, S35, S37, S38, and Fyv4) appear lost in euglenids yet retained in trypanosomes (Zíková et al. 2008), whereas 10 subunits (L1, L6, L18, L38, L56, S2, S3, S7, S13, and S28) are present solely in the *E. gracilis* mitoribosome (supplementary table 7, Supplementary Material online). These compositional distinctions potentially underpin major differences in protein synthesis between euglenids and kinetoplastids.

Kinetoplastids also contain a uniquely large number of novel mitoribosomal subunits, indicating expansions that occurred later in euglenozoan evolution (Desmond et al. 2011). Based on structural analysis, many have been reclassified as core or accessory subunits (Ramrath et al. 2018), with only one found outside kinetoplastids, in *Naegleria gruberi* (Desmond et al. 2011). Of the 79 kinetoplastid-specific subunits, 15 were recovered in the *E. gracilis* mitoproteome, with a further 20 predicted within the transcriptome (supplementary table 7, Supplementary Material online). This finding rules out the possibility that these subunits developed in response to a parasitic lifestyle, as previously proposed (Desmond et al. 2011). Of shared mitoribosomal proteins, a greater percentage of predicted small ribosomal subunit proteins (7/12) were recovered in vivo when compared with the large subunit (7/23). One ribosomal protein identified in *T. brucei* that localizes to both large and small subunits (Zíková et al. 2008) was also recovered here (supplementary table 7, Supplementary Material online).

A total of 18 mitoribosomal proteins, which did not match previously defined subunit groups were present either in the mitoproteome or predicted in silico and are therefore potential novel *E. gracilis* subunits. Twelve and four of them were predicted to reside in large and small mitoribosomal subunits respectively, whereas two were unclassified (supplementary table 7, Supplementary Material online). Finally, eight putative homologs of mitoribosomal proteins were identified in *E. gracilis*. Given high divergence generally encountered among this category of proteins (Petrov et al. 2019), and the fact that these eight proteins with *E*-values above the stated cutoff were detected by mass spectrometry, we classify them as putative mitoribosomal components (supplementary table 7, Supplementary Material online). Our results suggest that considerable mitoribosome protein expansion occurred already in the common euglenozoan ancestor (Desmond et al. 2011).

### Aminoacyl-tRNA Synthetases

Aminoacyl-tRNA synthetases (aaRSs) are responsible for attaching amino acids to their cognate tRNAs (de Duve 1988). Two classes exist, which differ in their domain architecture, ATP binding conformation and modes of aminoacylation (Burbaum and Schimmel 1991). Along with direct aminoacylation carried out by aaRSs, some archaea, bacteria, and chloroplasts lacking GlnRS and AsnRS enzymes possess indirect aminoacylation pathways, where a nondiscriminating GluRS and tRNA-dependent amidotransferase (AdT) carries out biosynthesis of Gln-tRNA^Gln^ and Asn-tRNA^Asn^ (Schön et al. 1988). We identified 46 transcripts encoding all class I and II aaRSs in *E. gracilis* (supplementary table 8, Supplementary Material online). Each of the identified proteins had at least one paralog (or isoform) with predicted mitochondrial localization, except ArgRS and TrpRS, likely missing in the mitochondrion, whereas MetRS, IleRS, and GluRS appear to be dually targeted to the mitochondrion and chloroplast (supplementary table 8, Supplementary Material online). Moreover, each of these aaRSs has an additional paralog of low sequence identity (varying from 24% to 44%) targeted exclusively to the mitochondrion. The identification of several aaRSs in the mitochondrial fraction suggests that some of them might have unconventional functions (Francklyn et al. 2002). The presence of GlnRS and AsnRS along with the absence of clear homologs of AdTs in any of the publicly available *E. gracilis* transcriptomes indicates that only the direct aminoacylation pathway is functional in both organelles and cytoplasm. This is in agreement with recent identification of a plastidial version of GlnRS in the nonphotosynthetic *Euglena longa* (Záhonová et al. 2018).

### Amino Acid Metabolism

We identified most of the biosynthesis pathways for 11 amino acids: valine, leucine, isoleucine, aspartate, cysteine, glutamate, glutamine, glycine, serine, proline, and tyrosine (supplementary fig. 6, Supplementary Material online), suggesting that the repertoire of amino acid biosynthesis enzymes is substantially richer in the mitochondrion of *E. gracilis* than in its plastid (Novák Vanclová et al. 2020). Although hydroxymethyltransferase catalyzes serine and glycine interconversion in *E. gracilis* mitochondria, glutamate is synthesized from 2-oxoglutarate by the action of aspartate aminotransferase and can be subsequently converted to glutamine or proline (supplementary fig. 6, Supplementary Material online), and aspartate can be synthesized from pyruvate using pyruvate carboxylase and aspartate aminotransferase.

The biosynthesis of valine and leucine from pyruvate does not appear possible in the *E. gracilis* mitoproteome, since only one enzyme of the pathway, 2-isopropylmalate synthase, is present. Another branched-chain amino acid, isoleucine, can be obtained in a five-step reaction from threonine, whereas cysteine is synthesized de novo from serine, and tyrosine is formed from phenylalanine by phenylalanine hydroxylase (supplementary fig. 6, Supplementary Material online).

The use of amino acids as an energy source in mitochondrion in *E. gracilis* is limited to the ability to degrade valine, leucine, isoleucine, glutamate, glycine, proline, aspartate, alanine, and possibly cysteine (supplementary table 8, Supplementary Material online). Almost all enzymes of the branched-chain amino acids degradation pathway leading to formation of acetyl-CoA, in the case of leucine and isoleucine, and succinyl-CoA or valine, were identified. Proline is converted to glutamate in a two-step reaction carried out by proline dehydrogenase and δ-1-pyrroline-5-carboxylate dehydrogenase, and glutamate can then be degraded either to α-ketoglutarate and ammonia or to succinate. In contrast to human mitochondria, it appears that cysteine degradation in the organelle of *E. gracilis* leads to pyruvate formation. We identified a putative homolog of thiosulfate/3-mercaptopyruvate sulfurtransferase, catalyzing the second step of this process. Cysteine aminotransferase was not detected, however, leaving the possibility that the initial reaction of the process is catalyzed by aspartate aminotransferase, similar to some bacteria (Andreeßen et al. 2017). Although glycine is oxidatively cleaved by the glycine cleavage system, the saccharopine pathway of lysine degradation appears to be nonfunctional, since only three enzymes out of nine were identified (supplementary table 8, Supplementary Material online).

### Sulfate Assimilation

A remarkable feature of the *E. gracilis* mitochondrion is the ability to assimilate and metabolize sulfate (Saidha et al. 1988). This represents an exceptionally rare feature among eukaryotes, as the vast majority employ the sulfate assimilation pathway in either resident plastids or the cytosol. Some of the enzymes involved in this process have a plastid origin, but over time have been retargeted to the mitochondria, presumably after their genetic incorporation into the nucleus (Patron et al. 2008). Components of the sulfate assimilation pathway have additionally been found in the mitosomes of *Entamoeba histolytica*, though these are thought to have been acquired independently from ε-proteobacteria and other sources (Mi-ichi et al. 2009). This pathway is important for the generation of sulfur-containing amino acids such as cysteine, methionine, and various other metabolites, which supply necessary sulfur for the Fe-S cluster generation in euglenids (Saidha et al. 1988). Except for sulfate permease and cysteine synthase, all enzymes for sulfate assimilation were identified by proteomics (supplementary table 7 and file 3, Supplementary Material online).

### Fe-S Cluster Biosynthesis

Iron-sulfur (Fe-S) cluster biosynthesis provides essential and ubiquitous cofactors for many proteins (Lill 2009). In most eukaryotes, the first step, referred to as ISC biosynthesis, is located in the mitochondrion (Braymer and Lill 2017). Among euglenozoans, Fe-S cluster assembly is best studied in *T. brucei*, whereas in the euglenids and diplonemids, the pathway remains unexplored (Ali and Nozaki 2013; Peña-Diaz and Lukeš 2018). We find conservation of all predicted central components of this pathway, with some notable expansions.

**Fig. 5.**
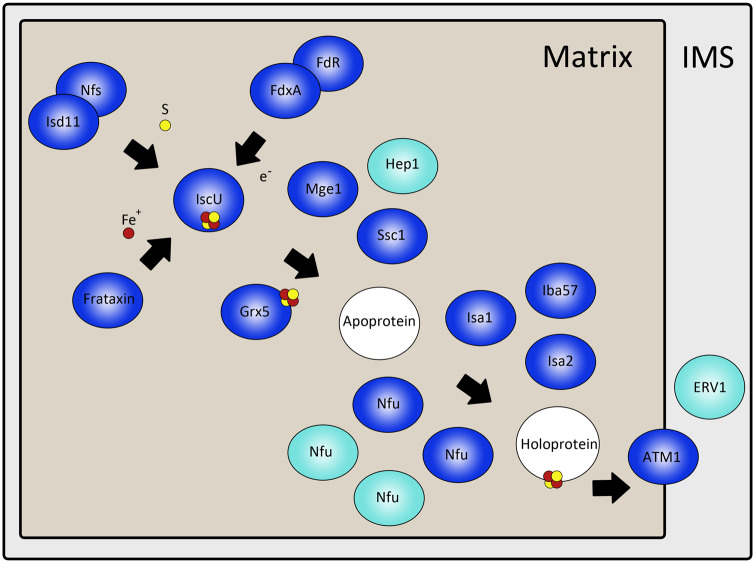
ISC biosynthesis in *Euglenac gracilis* mitochondrial matrix and IMS. Blue for proteomically confirmed sequences, light blue for sequences identified in transcriptome, and white for proteins requiring ISC insertion for functionality.

The ISC pathway consists of three central steps, starting with Fe-S cluster assembly on a scaffold protein IscU. Sulfur is donated by cysteine desulfurase NfsI and Isd11, after the cluster is reduced by ferredoxin in coordination with ferredoxin reductase (Ollagnier-de-Choudens et al. 2001). Iron (II) cation is imported into the matrix via mitochondria carrier protein 17 (Braymer and Lill 2017), where it is donated to the scaffold with the assistance of frataxin (Bridwell-Rabb et al. 2014). In *E. gracilis*, two ferredoxin orthologs were identified (fig. 5), corresponding to *T. brucei* ferredoxin A and B (Changmai et al. 2013). The second step involves detachment of the Fe-S cluster from the IscU scaffold. Grx5, Mge1, Ssc1, and Hep1 assist as chaperones, transporting the cluster to the relevant apoprotein (Maio et al. 2014). Peptides for Grx5, Mge1, and Ssc1 were identified at the protein level, with Hep1 being identified only in silico (fig. 5). The final step involves alternative scaffold proteins Isa1 and Isa2, with assistance from Iba57, which insert the Fe-S clusters into various apoproteins (Sheftel et al. 2012). All three sequences were confirmed at a protein level (fig. 5).

Depending on protein specificity, various factors are subsequently required for holoprotein maturation, such as Nfu, involved in maturation of respiratory complexes I and II components (Lukeš and Basu 2015). In *E. gracilis*, four Nfu factors are identifiable from the transcriptome, with two being recovered as peptides (fig. 5 and supplementary table 7, Supplementary Material online). One Nfu homolog was initially rejected due to insufficient enrichment but reintegrated based on functional prediction. Most eukaryotes contain only a single Nfu protein, whereas expansion into three and four Nfus occurred in *T. brucei* and *Leishmania* spp., respectively, of which most are mitochondrially localized (Benz et al. 2016). *Arabidopsis thaliana* contains a higher number of Nfu paralogs, but most are localized in the plastid (Léon et al. 2003). By contrast, in *E. gracilis* both Nfu proteins show mitochondrial enrichment when compared with the chloroplast (supplementary table 4, Supplementary Material online). The Fe-S clusters bound for export out of the mitochondrion are transported by Atm1 and Erv1 (Braymer and Lill 2017), both being identified in silico, and Atm1 also at the protein level (fig. 5).

### Fatty Acid Metabolism

Under anaerobic conditions, the mitochondrion of *E. gracilis* can employ acetyl-CoA as a terminal electron acceptor and synthesize fatty acids in a malonyl-independent manner, enabling net ATP production (Hoffmeister et al. 2005). Acetyl-CoA is first produced from the catalysis of pyruvate, mediated by an oxygen-sensitive pyruvate:NADP+ oxidoreductase (PNO) complex (Zimorski et al. 2017). Both α and β subunits of the PNO complex were recovered at a protein level (supplementary table 7, Supplementary Material online). Although the β subunit showed low mitochondrial enrichment, likely due to the oxygen-sensitive nature of PNO (Rotte et al. 2001) and the aerobic conditions of this study, it was included based on strong prediction criteria. The synthesis of fatty acids involves reversal of the β-oxidation of acetyl-CoA pathway. Several enzymes involved in this process can work in both directions (Zimorski et al. 2017). In *E. gracilis*, acetyl-CoA is first condensed by acetyl-CoA-C-acetyltransferase, then reduced via β-hydroxyacyl-CoA and dehydrated by enoyl CoA hydratase. The final step is mediated by unique *trans-*2-enoyl CoA reductase, as the enzyme responsible for the reverse reaction (acyl-CoA dehydrogenase) operates catalytically in one direction (Zimorski et al. 2017). The activity of *trans-*2-enoyl CoA reductase produces elongated fatty acyl-CoA as an end product, which is reduced to alcohol, esterified, and deposited in the cytosol as wax esters (Hoffmeister et al. 2005). Upon returning to aerobic conditions, in the mitochondrion, these wax esters can be converted back to acetyl-CoA (Zimorski et al. 2017).

We have detected peptides for all four enzymes required for fatty acid synthesis, whereas oxidative acyl-CoA dehydrogenase was identified only by a homology search. Although *E. gracilis* was cultivated in aerobic conditions, *trans-*2-enoyl CoA reductase, which is exclusively used for anaerobic fatty acid synthesis, was detected (supplementary table 7, Supplementary Material online). Its expression under aerobic conditions is in agreement with previous studies and reflects the adaptability of *E. gracilis* for environments deprived of oxygen (Hoffmeister et al. 2004). All proteins involved in long-chain acyl-CoA import into the mitochondrion, as well as components for odd-numbered fatty acid synthesis (excepting fumarate reductase and propionyl-CoA subunits), were also proteomically recovered (supplementary file 3, Supplementary Material online).

### In Silico Mitoproteome of *Eutreptiella gymnastica*

To gain a better understanding of mitoproteome evolution within euglenida and to resolve the seemingly unique features of *E. gracilis*, we compared the verified mitoproteome with the publicly available transcriptome of fellow photosynthetic euglenid *Eut. gymnastica*, the outcome of which yielded 1,162 transcripts corresponding to 900 ortholog groups (supplementary table 9, Supplementary Material online). Six hundred and twenty of these *Eut. gymnastica* transcripts were orthologous to functionally uncategorized sequences of *E. gracilis*, which suggests shared heritage to a large expansion of these unknown genes within the ancestor of photosynthetic euglenids.

**Fig. 6.**
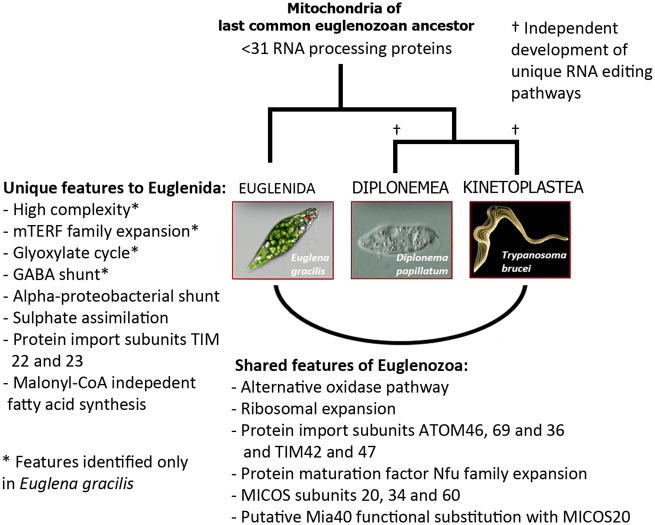
Evolutionary schematic showing unique mitochondrial features to euglenida, with features observed only in *Euglenac gracilis* denoted by *. Common features discovered in this study with the *Trypanosoma brucei* mitoproteome are listed, which were likely present in the mitochondria of the last euglenozoan common ancestor. Over 30 RNA editing enzymes appear to be present in this ancestor, which led to development of different RNA editing pathways in diplonemid and kinetoplastid lineages, denoted by.

Although analysis of the *E. gracilis* transcriptome identified an extensive 192 mTERF factors identified in *E. gracilis*, no orthologs could be identified from *Eut. gymnastica*. Comparison with the transcriptome of *Eut. gymnastica* indicated conservation of all components of the alpha-proteobacterial shunt, whereas only orthologs to GABA transaminase of the GABA shunt could be identified (fig. 6 and supplementary table 9, Supplementary Material online). Components necessary for the glyoxylate cycle were not identified, suggesting this pathway may be unique to *E. gracilis*. Interestingly, Hidden Markov Model searches identified a sequence above the detection threshold to subunit E2 of oxaglutarate dehydrogenase (supplementary table 9, Supplementary Material online), suggesting that the conventional TCA pathway could in fact be functional in *Eut. gymnastica*.

The recovery of inner membrane translocation pores TIM22 and TIM23 in *E. gracilis* represents a first for Euglenozoan mitochondria, and their identification within the transcriptome of *Eut. gymnastica* suggests they are distributed throughout euglenida as well (fig. 6 and supplementary table 9, Supplementary Material online). Although four protein maturation factors were identified for *E. gracilis*, no Nfu factors could be identified from the *Eut. gymnastica* transcriptome, suggesting that this high Nfu number may be confined to *E. gracilis*.

Orthologs to the majority of sulfate assimilation enzymes (seven of nine) were additionally identified in the *Eut. gymnastica* transcriptome (supplementary table 9, Supplementary Material online), suggesting that the mitochondrial retargeting of sulfate occurred in the common ancestor of these two species. Additionally, orthologs for the majority of components for fatty acid synthesis and import (nine of seventeen) were also identified, including crucial enzyme *trans*-2-enoyl CoA reductase (supplementary table 9, Supplementary Material online), suggesting that malonyl-independent fatty acid synthesis is a conserved feature in euglenids (fig. 6).

## Conclusions

The exceptionally rich mitoproteome of *E. gracilis* can be partially attributed to extensive duplications, including mitoribosomal subunits and the AOX pathway as well as core metabolic pathways where multiple paralogs may indicate considerable flexibility. However, the main contributing factor to this unique complexity is the exceptionally large fraction of proteins with unknown function. Additionally, over 700 predicted proteins such as the extensive mTERF family still require validation through additional functional studies. Although we report the identification of various anaerobic enzymes such as *trans-*enoyl-reductase and oxygen-sensitive PNO, it is likely that many strictly anaerobic proteins have eluded detection due to the aerobic nature of the extracted mitochondria. Subsequently, a study of the organelle from the anaerobically grown *E. gracilis* is a logical successive step.

Characterization of the *E. gracilis* mitoproteome represents an important step in our understanding of the uniquely rich evolution of this organelle in different euglenozoan lineages and enables the prediction of mitochondrial traits present in the euglenozoan common ancestor. Such traits include newly identified mitoribosomal and MICOS subunits, protein import machinery, respiratory proteins, components of the Fe-S cluster biosynthesis as well as the prominent absence of RNA editing and processing machinery (fig. 6). Combined, the mitoproteome of *E. gracilis* demonstrates an unparalleled protein count, which may reflect the influence of the co-occurring plastid, and reveals a remarkable metabolic flexibility and adaptability.

## Materials and Methods

### Cell Culture and Isolation of Mitochondria

*Euglena gracilis* strain Z1 cells were axenically cultured in total volume of 600 ml in Hutner’s medium at 27 °C in aerobic conditions under permanent light (10 µm/m^−2^ s^−1^) with constant shaking at 150 rpm until they reached exponential growth (1.5–2 × 10^6^ cells/ml). Cells were collected by centrifugation at 800 × g for 10 min and resuspended in SHE buffer (250 mM sucrose, 10 mM HEPES, 1 mM EDTA, pH 7.3) supplemented with 0.4% fatty acid–free bovine serum albumin. All the following steps were performed on ice. *Euglena* cells were disrupted by sonication at 80% power using a thick 19.5-mm probe (ultrasonic homogenizer 3,000; Biologics, Inc.). Sonication was performed in six 10-s pulses cycles with 2-min breaks. The sonicate was centrifuged for 15 min at 800 × g and 4 °C and the resulting supernatant centrifuged for 15 min at 8,500 × g at 4 °C. The pellet was resuspended in 3 ml of STM buffer (250 mM sucrose, 20 mM Tris–HCl, 2 mM MgCl_2_, pH 8.0) with 40 U of DNase I (Thermo Scientific) and incubated on ice for 30–60 min. DNase-treated lysate (5 ml) was loaded on top of a discontinuous sucrose density gradient. The gradient was prepared by layering decreasingly dense sucrose solutions upon one another from 2.0, 1.75, 1.5, 1.25, 1, to 0.5 M (5 ml each) and centrifuged in a SW-28 rotor at 87,000 × g at 4 °C for 4.5 h (L8-M, Beckman).

The mitochondrial fraction was separated from chloroplast and peroxisome fractions, located at the interface of 1.7 and 1.5 M sucrose layers and collected with a syringe. To remove excess sucrose, mitochondria were washed twice in SHE buffer. The final pellet was gently resuspended with a cutoff pipette tip in 500–1,000 μl of SHE buffer supplemented with 0.4% bovine serum albumin, and then spun for 30 min at 16,000 × g at 4 °C and stored at −80 °C.

### Mass Spectrometry–Based Protein Identification and Quantification

Samples were sonicated in NuPAGE LDS sample buffer (Thermo Sci.) containing 2 mM dithiothreitol and separated on a NuPAGE Bis-Tris 4–12% gradient polyacrylamide gel (Thermo) under reducing conditions. The sample lane was divided into eight slices that were excised from the Coomassie-stained gel, destained, and subjected to tryptic digest and reductive alkylation. The treated fractions were subjected to liquid chromatography tandem mass spectrometry on an UltiMate 3000 RSLCnano System coupled to a Q exactive mass spectrometer (Thermo Sci.). Mass spectra were analyzed using MaxQuant V. 1.56 (Cox and Mann 2008) and by searching the predicted translated transcriptome of Ebenezer et al (2019). Minimum peptide length was set to six amino acids, isoleucine and leucine were considered indistinguishable and false discovery rates of 0.01 were calculated at the levels of peptides, proteins, and modification sites based on the number of hits against the reversed sequence database. Ratios were calculated from label-free quantification intensities using only peptides that could be uniquely mapped to a given protein. If the identified peptide sequence set of one protein contained the peptide set of another protein, these two proteins were assigned to the same protein group. *P* values were calculated applying *t*-test based statistics using Perseus (Tyanova et al. 2016). Protein sequences (2,704) were initially identified in the mitochondrial fraction which satisfied these criteria (supplementary table 1, Supplementary Material online). Proteomics data have been deposited to the ProteomeXchange Consortium by the PRIDE (Vizcaíno et al. 2016) partner repository with the data set identifier PXD014767.

### Identification of Mitochondrially Encoded Proteins

Proteins encoded in the *E. gracilis* mitochondrial genome, determined by Miranda-Astudillo et al. (2018) and Dobáková et al. (2015) (supplementary table 2, Supplementary Material online), were missing from the translated transcriptome, except for cox3. The MaxQuant LFQ analysis was repeated using this search database composed of seven protein sequences. The resulting quantifications of four additional mitochondrial-encoded proteins were included in the candidate data set.

### Reference Mitoproteome Comparison

Proteins of the mitochondrial fraction of *E. gracilis* were compared with data sets containing the mitoproteomes of *A. thaliana*, *M. musculus*, *S. cerevisiae*, and *T. brucei* (supplementary table 3, Supplementary Material online). For *S. cerevisiae*, 1,010 mitochondrial proteins were selected using a combination of the following criteria: 1) Gene ontology term “mitochondrion” assigned to the protein and 2) annotation as mitochondrial assigned manually based on experimental studies, or identified as mitochondrial in ≥2 high-throughput analyses, or one high-throughput analysis plus computational evidence. The data set for *A. thaliana* is composed of 722 mitochondrial proteins based on Lee et al. (2013) (supplementary table 3, Supplementary Material online). Mitochondrial proteins of *M. musculus* were downloaded from the Mitocarta database v.2.0, whereas for *T. brucei*, mitochondrial importome comprising 1,120 proteins identified using ImportOmics method was used. For each species within the data set, only one representative protein isoform for each gene was included. *Euglena gracilis* sequences were included in accepted mitoproteome if an orthologous sequence was detected in at least one of the mitoproteomes (994 sequences) (supplementary table 4, Supplementary Material online).

Predicted proteins from three available transcriptomes (O’Neill et al. 2015; Yoshida et al. 2016; Ebenezer et al. 2019) were analyzed via TargetP 1.1 for predicted organellar localization (Emanuelsson et al. 2000). Transcript open reading frames starting with methionine and predicted to be imported into the mitochondrion in the majority of transcriptomes were considered as mitoproteome components (77 sequences). Mitochondrial fraction proteins were additionally compared against in silico predicted *E. gracilis* mitoproteome (Ebenezer et al. 2019), including any predicted sequences that were identified by liquid chromatography tandem mass spectrometry from the mitochondrial fraction (552) (supplementary table 4, Supplementary Material online). Proteins with mitochondria to chloroplast and mitochondria to whole cell ratios >1, indicating mitochondrial enrichment compared with the chloroplast and whole cell, were considered as mitochondrial (1,543) (supplementary table 5, Supplementary Material online). Mitochondrial-to-chloroplast enriched sequences were assigned confidence values of the logarithmic enrichment ratio of the mitochondrial versus chloroplast preparation (log 10Mt/Cp), and sorted into groups of 0.0–1.0, >1.0 (supplementary table 5, Supplementary Material online).

### Functional Characterization and Mitochondrial Protein Families

Proteins considered as candidate mitoproteome components were annotated automatically using BLAST against the NCBI nonredundant protein database (August 2016 version). These annotations were checked manually consulting UniProt database and putative homologs inferred using OrthoFinder 2.2.7 as necessary (Emms and Kelly 2015). Proteins annotated as having roles in specific metabolic pathways or molecular complexes were sorted manually into 16 custom-defined categories roughly based on the KEGG pathway classification. Twenty-four proteins were removed from the mitoproteome based on functional annotation showing clear nonmitochondrial function as well as a lack of orthologs in any reference mitoproteomes. Annotations of the predicted mitoproteome of *E. gracilis* (Ebenezer et al. 2019) were first compared with the 2,704 sequences of mitochondrial fraction (supplementary table 1, Supplementary Material online) to determine functional proteins that were present (552). Functional protein families determined were then complemented via manual search for functionally annotated members from the experimentally verified mitoproteome. Missing key sequences from established mitochondrial protein families were then investigated with a combination of BLAST and Hidden Markov Model searches.

### Hidden Markov Models

Sequences underwent ClustalW alignment via Mega10.0.5 (Kumar et al. 2018) and were searched using HMMER software against proteins generated primarily from the *E. gracilis* transcriptome (O’Neill et al. 2015; Yoshida et al. 2016; Ebenezer et al. 2019), which were accepted with an *E*-value of 10^−3^ or lower and a bias score lower than 1. Topogenesis of β-barrel protein TOB38, Sorting and assembly machinery SAM37, ATOM subunits 19, 14, 12, and 11, peripheral ATOM36, TIM8, 11, 13, 40, 47, 54, 62, tiny TIMs, MICOS subunits 10-1, 10-2, 16, 17, 20, 32, 34, 40, and 60, Hsp10, Nfu’s, and Coq7 were queried for with HMM profiles constructed using all available functionally annotated kinetoplast sequences from TriTryp website (Aslett et al. 2010). IMP1 and IMP2 were also determined using a spread of sequences from Amoebozoa, Archaeplastida, and Alveolata. Presequence translocated–associated motor subunits 17 and 18 were searched for using a selection of metazoan sequences. Metaxin sequences were searched for using a combination of metazoan, plants, and protist sequences. TIM18 and TIM54 (both nontrypanosome), mammalian MICOS19/25, Coq6, and TOM22 were searched for using a selection of opisthokont sequences. Proteins identified as MICOS hits were analyzed with TMHMM (Kroph and Rapacki 2013) and Coiled Coils (Combet et al. 2000) to identify structural features.

### BLAST Searches

Mitochondrial ribosomes, subunits of respiratory complexes, and protein import machinery (TIM11, TIM13 RHOM 1, RHOM 2, TINY TIM, Oxa2, and TIM42) were searched using BLAST against the assembled *E. gracilis* transcriptome (Ebenezer et al. 2019) with *T. brucei* sequences as the query. Sequences with an *E*-value of 10^−3^ or less were accepted. Mitochondrial ribosomal subunits from databases of *T. brucei* (Zíková et al. 2008; Desmond et al. 2011; Ramrath et al. 2018), and *Diplonema papillatum* (our unpublished data) were employed for BLAST searches. For mitochondrial ribosomal subunits not present in excavates, sequences from *Homo sapiens* and *S. cerevisiae* were used as queries (Desmond et al. 2011). mTERF-like proteins of *A. thaliana* and *H. sapiens* (Kleine and Leister 2015) were used for BLAST searchers. Protein sequences of *E. gracilis* respiratory complex subunits from previous studies (Perez et al. 2014; Miranda-Astudillo et al. 2018) were used as queries.

### Survey of aaRSs and Amino Acid Metabolism

The search for aaRSs and related enzymes (tRNA-dependent AdTs) was performed using annotated sequences of *H. sapiens*, *Plasmodium falciparum*, and *A. thaliana* as queries and all transcripts and predicted proteins of *E. gracilis* as database for BLAST searches with an *E*-value cutoff of 10^−20^. For AdTs (Pfam models PF01425, PF02686, and PF02934), additional HMM-based searches were performed using HMMER package v.3.1 (Eddy 2009) and predicted proteins from this study, as well as publicly available *E. gracilis* transcriptomes (O’Neill et al. 2015; Yoshida et al. 2016). InterProScan, Pfam, and BlastKoala were used to facilitate functional annotation of the hits (Kanehisa et al. 2016; Kanehisa 2017; El-Gebali et al. 2019; Mitchell et al. 2019). NLStradamus, SeqNLS, and NLS-Mapper were employed for nuclear localization signals prediction (Ba et al. 2009; Kosugi et al. 2009; Lin and Hu 2013). Reconstruction of mitochondrial amino acid metabolic pathways was performed with KEGG Mapper v.2.8 following KEGG IDs assignment to putative mitochondrial proteins using BlastKoala (Kanehisa et al. 2016).

### Comparison to *Eut. gymnastica*

The experimentally determined *E. gracilis* mitoproteome was compared with the translated transcriptome of *Eut. gymnastica* strain NIES-381 with Orthofinder 2.2.7 (Emms and Kelly 2015). The *Eut. gymnastica* transcriptome additionally underwent Hidden Markov Model searches (with previously described parameters) against all available kinetoplastid sequences from TriTrypDB for the oxoglutarate decarboxylase complex subunits E1 and E2.

## Supplementary Material

msaa061_Supplementary_Data
