# Supplementary material for: A Uniquely Complex Mitochondrial Proteome from Euglena gracilis
Source: Mol Biol Evol. 2020 Apr 5;37(8):2173–91. doi: 10.1093/molbev/msaa061 (PMC7403612; doi:10.1093/molbev/msaa061)
Supplement: msaa061_Supplementary_Data [file msaa061_supplementary_data.zip › msaa061-suppl_data/Suppl. File 3..pdf]

The Sulphate assimilation pathway

The sulphate assimilation pathway involves uptake of sulphate, into the mitochondria, and is activated before being reduced, allowing for synthesis of cysteine. Sulphate is transported into the mitochondria by sulphate permease and adenylated by sulphate adenylyl-transferase, then phosphorylated by adenylyl-sulphate kinase. Subsequently, it is reduced by adenylyl-sulphate reductase to sulphite and converted by the two-part complex sulphite reductase flavoprotein to sulphide (Patron et al. 2008). This sulphide is then conjugated with serine via the enzyme serine O-acetyl-transferase. The intermediate product O-acetyl serine is finally converted to cysteine by cysteine synthase (Patron et al. 2008). Except for sulphate permease and cysteine synthase, all enzymes for sulphate assimilation were identified by proteomics (Suppl. Table 7).

Ubiquinone synthesis

Ubiquinone represents a key molecule in the respiratory chain; its ability to be reduced and oxidised allows it to transfer electrons between complexes (Kawamukai 2016). Ubiquinone synthesis involves coupling the benzoquinone ring 4-hydroxybenzoate with an isoprenoid side chain, mediated by the enzyme Coq2 (Stefely and Pagliarini 2017). The aromatic ring undergoes a series of modifications, first involving O-methylation, hydroxylation, C-methylation, hydroxylation and O-methylation performed by Coq3, Coq6, Coq5, Coq7 and Coq3 once more, respectively, resulting in functional ubiquinone-N (Kawamukai 2016).

The *E. gracilis* mitochondrion shows a slightly diverged pathway for ubiquinone synthesis. Orthologues of all enzymes of the ubiquinone pathway except Coq7 were identified (Ebenezer et al. 2019), while Coq3, Coq5 and Coq6 were recovered at the protein level (Suppl. Table 7). The various Coq proteins have been shown to function in an interdependent manner in yeast, where the lack of one protein results in others being degraded (Xie et al. 2012), making the absence of Coq7 peculiar. It is possible that the *E. gracilis* Coq7 radically diverged to the point that it cannot be identified, or that another enzyme performs the final hydroxylation step.

Long chain import and odd-numbered chain fatty acid synthesis

Import of long chain acyl-CoA from the cytosol to the mitochondria is mediated by carnitine O-palmitoyltransferases 1 and 2 found in the outer and inner membrane, respectively. Since acyl-CoA cannot cross membranes in its active form, carnitine O-palmitoyltransferase 1

catalyses the formation of acyl-carnitine from acetyl-CoA and carnitine, enabling its *trans*-membrane transport. Once in the mitochondrial matrix, this reaction is reversed via carnitine O-palmitoyltransferase 2, yielding carnitine and active acyl-CoA (Jogl et al. 2004). Both transferases were recovered in *E. gracilis*, strongly indicating capacity to import acyl-CoA into the mitochondrion (Suppl. Table 7). Synthesis of long chain acyl-CoA before mitochondrial import is catalysed by membrane-anchored long-chain acyl-CoA synthetase (Hisanaga et al. 2004), which was also recovered.

For the synthesis of odd-numbered fatty acid chains, the mitochondrion makes use of propionyl-CoA, as a starting template (Müller et al. 2012). Propionyl-CoA is generated through the methylmalonyl-CoA pathway, where mitochondrial malate is catalysed to fumarate using fumarate hydratase, in the reverse direction to that encountered in the TCA cycle (Zimorski et al. 2017). Next, fumarate is further converted to succinate by the membrane-bound fumarate reductase via succinyl-CoA, which is converted to methylmalonyl-CoA by the action of dual enzymes methylmalonyl-CoA mutase and epimerase, before being finally catalysed to propionyl-CoA by the propionyl-CoA carboxylase complex (Miyamoto et al. 2010; Müller et al. 2012). Both the alph and beta □ components of propionyl-CoA carboxylase complex are proteomically present, with two sequences showing similarity to the subunit (Suppl. Table 7). With the exception of fumarate reductase and propionyl-CoA subunits, all components of the methylmanoyl, for which only *in silico* evidence is available, all other components of the methylmalonyl-CoA pathway, as well as fumarate hydratase and succinyl-CoA synthetase subunits were confirmed at a protein level (Fig. 5) (Suppl. Table 7).

List of references

Ebenezer TE, Zoltner M, Burrell A, Nenarokova A, Vanclová A, Prasad B, Soukal P, Santana-Molina C, O'Neill E, Nankissoor NN et al. 2019. Transcriptome, proteome and draft genome of *Euglena gracilis*. BMC Biology 17:11.

Hisanaga Y, Ago H, Nakagawa N, Hamada K, Ida K, Yamamoto M, Hori T, Arii Y, Sugahara M, Kuramitsu S et al. 2004. Structural basis of the substrate-specific two-step catalysis of long chain fatty acyl-CoA synthetase dimer. Journal of Biological Chemistry 279(30):31717-31726.

Jogl G, Hsiao YS, Tong L. 2004. Structure and function of carnitine acyltransferases. Carnitine: the Science Behind a Conditionally Essential Nutrient 1033:17-29.

Kawamukai M. 2016. Biosynthesis of coenzyme Q in eukaryotes. Bioscience Biotechnology and Biochemistry 80(1):23-33.

Miyamoto E, Tanioka Y, Nishizawa-Yokoi A, Yabuta Y, Ohnishi K, Misono H, Shigeoka S, Nakano Y, Watanabe F. 2010. Characterization of methylmalonyl-CoA mutase involved in the propionate photoassimilation of *Euglena gracilis* Z. Archives of Microbiology 192(6):437-446.
Müller M, Mentel M, van Hellemond JJ, Henze K, Woehle C, Gould SB, Yu RY, van der Giezen M, Tielens AGM, Martin WF. 2012. Biochemistry and Evolution of Anaerobic Energy Metabolism in Eukaryotes. Microbiology and Molecular Biology Reviews 76(2):444-495.
Patron NJ, Durnford DG, Kopriva S. 2008. Sulfate assimilation in eukaryotes: fusions, relocations and lateral transfers. BMC Evolutionary Biology 8:14.
Stefely JA, Pagliarini DJ. 2017. Biochemistry of Mitochondrial Coenzyme Q Biosynthesis. Trends in Biochemical Sciences 42(10):824-843.
Xie LTX, Ozeir M, Tang JY, Chen JY, Jaquinod SK, Fontecave M, Clarke CF, Pierrel F. 2012. Overexpression of the Coq8 Kinase in *Saccharomyces cerevisiae* coq Null Mutants Allows for Accumulation of Diagnostic Intermediates of the Coenzyme Q(6) Biosynthetic Pathway. Journal of Biological Chemistry 287(28):23571-23581.
Zimorski V, Rauch C, van Hellemond JJ, Tielens AGM, Martin WF. 2017. The Mitochondrion of *Euglena gracilis*. In: Schwartzbach SD, Shigeoka S, editors. Euglena: Biochemistry, Cell and Molecular Biology. Cham: Springer. p. 19-37.
