# Supplementary material for: A Uniquely Complex Mitochondrial Proteome from Euglena gracilis
Source: Mol Biol Evol. 2020 Apr 5;37(8):2173–91. doi: 10.1093/molbev/msaa061 (PMC7403612; doi:10.1093/molbev/msaa061)
Supplement: msaa061_Supplementary_Data [file msaa061_supplementary_data.zip › msaa061-suppl_data/Suppl. Table 3..pdf]

**Species**	**Mitochondrial proteome source**	**Number of proteins**	**Comments**
Euglena gracilis	Our study	2704	All proteins found in the mitoproteome fraction. The protein IDs were taken from Supplementary table supplementary table 1. Proteins corresponding to the reverse translated genes, were not taken.
Trypanosoma brucei	Peikert et al., 2017	1231	All nuclear-encoded proteins from file "ATOM40_pure_importome_list_1120.txt", TriTrypDB version 8.1. Proteins, corresponding to the reverse translated genes, were not taken.
Arabidopsis thaliana	Lee et al., 2013	724 (722- found two duplicated proteins in the file)	504 from list B + 222 from list C.<br>AT5G54770 is in both lists, AT5G15910 is duplicated in list B.<br>Protein sequences were taken from Apaport11 (TAIR), "represent active gene model" unless other isoform specified. For AT2G46540 was used FGENESH2_KG.4__2874__AT2G46540.1.1
Mus musculus	Calvo et al., 2015	1158	For each gen the longest or the first isoform from the file "Mouse.MitoCarta2.0.fasta" was used, obtained from MitoCarta2.0 site.
Saccharomyces cerevisiae	Saccharomyces Genome Database	1010	Proteins with GO Term "mitochondrion" from SGD were taken according to the following criteria: it was assigned from "manually curated" reference OR it has two or more other references, such as "high-throughput" or "computational". Protein sequences were obtained from "orf_trans_all.fasta" file from Genome Release 64-2-1. The full description is available in file "yeast_mito.xlsx". Proteins have '*' in the end of sequence

References

1. *Saccharomyces* Genome Database (SGD), https://www.yeastgenome.org
2. Calvo, S. E., Clauser, K. R., & Mootha, V. K. (2015). MitoCarta 2.0: an updated inventory of mammalian mitochondrial proteins. Nucleic Acids Research, 44 (October 2015), 1–7.
3. Conesa, A., Götz, S., García-Gómez, J. M., Terol, J., & Talón, M. (2005). Blast2GO : a universal tool for annotation, visualization and analysis in functional genomics research, 21(18), 3674–3676.
4. Dobakova, E., Flegontov, P., Skalicky, T., & Lukes, J. (2015). Unexpectedly streamlined mitochondrial genome of the euglenozoan Euglena gracilis. Genome Biology and Evolution, 7(12), 3358–3367.
5. Emanuelsson, O., Brunak, S., von Heijne, G., & Nielsen, H. (2007). Locating proteins in the cell using TargetP, SignalP and related tools. Nature Protocols, 2(4), 953–71.
6. Lee, C. P., Taylor, N. L., & Millar, A. H. (2013). Recent advances in the composition and heterogeneity of the Arabidopsis mitochondrial proteome. Frontiers in Plant Science, 4, 4.
7. Kanehisa, M., Sato, Y., Kawashima, M., Furumichi, M., and Tanabe, M. (2016). KEGG as a reference resource for gene and protein annotation. Nucleic Acids Res. 44, D457-D462
8. Moriya Y., Itoh M., Okuda S., Yoshizawa A., and Kanehisa M. (2007) KAAS: an automatic genome annotation and pathway reconstruction server. Nucleic Acids Research 35, W182-W185.
9. Ono, K., Kondo, M., Osafune, T., Miyatake, K., Inui, H., Kitaoka, S., Nakano, Y. (2003). Presence of glyoxylate cycle enzymes in the mitochondria of Euglena gracilis. Journal of Eukaryotic Microbiology, 50(2), 92–96.
10. Peikert, C.D., Mani, J., Morgenstern, M., Käser, S., Knapp, B., Wenger, C., Harsman, A., Oeljeklaus, S., Schneider, A., Warscheid, B., 2017. Charting organellar importomes by quantitative mass spectrometry. Nat. Commun. 8, 15272.
11. Perez, E., Lapaille, M., Degand, H., Cilibrasi, L., Villavicencio-Queijeiro, A., Morsomme, P., Cardol, P. (2014). The mitochondrial respiratory chain of the secondary green alga Euglena gracilis shares many additional subunits with parasitic Trypanosomatidae. Mitochondrion, 19, 338–349.
12. Saidha, T., Na, S. Q., Li, J. Y., & Schiff, J. A. (1988). A sulphate metabolizing centre in Euglena mitochondria. Biochemical Journal, 253(2), 533–539.
