# Supplementary material for: A Uniquely Complex Mitochondrial Proteome from Euglena gracilis
Source: Mol Biol Evol. 2020 Apr 5;37(8):2173–91. doi: 10.1093/molbev/msaa061 (PMC7403612; doi:10.1093/molbev/msaa061)
Supplement: msaa061_Supplementary_Data [file msaa061_supplementary_data.zip › msaa061-suppl_data/Suppl. Table 6..pdf]

	Cytosol	Acidocalcisome	Endosome	Peroxisome	Lysosome	Golgi	Nucleus	Surface
Protein ID **EG_transcript_**	**21524**	**2633**	**181**	**15991**	**10514**	**5712**	**53416**	**32527**
Annotation	Aldolase	VP1 Vacuolar proton translocating pyrophosphatase	CHC Clathrin heavy chain	PEX2 Peroxisomal biogenesis factor 2	Lysosomal aspartic protease	Coatomer subunit γ2	Histone H4	Articulin 80 kDa
**Ratio Mt/W**	**0.02**	**1.46**	**0.53**	**W only**	**7.4**	**0.01**	**0.02**	**0.04**
-log10p	3.3	0.5	1.9	2.2	2.6	3.3	3.6	4.4
Ratio Mt/CP	3.85	0.75	2.5	NaN	2.27	1.35	0.50	6.66
-log10p	1.5	0.6	2.8	NaN	1.5	0.3	0.8	2.9
Ratio CP/W	0.01	1.95	0.21	W only	3.30	0.01	0.04	0.01
-log10p	5.9	1.2	3.1	1.4	2.0	3.4	3.3	4.7
Unique peptides	19	31	90	1	7	40	20	13

	Chloroplast			Mitochondrion		
Protein ID **EG_transcript_**	**40006**	**158**	**25897**	**2112**	**23844**	**8912**
Annotation	light-harvesting complex I protein precursor LhcB5	Photosystem I P700 chlorophyll a apoprotein A1	light-harvesting complex I protein precursor Lhca2	Pyruvate dehydrogenase [NADP(+)], mitochondrial	ubiquinol-cytochrome c reductase iron-sulfur subunit	F-type H+-transporting ATPase subunit beta
**Ratio Mt/W**	**0.2**	**0.10**	**NaN**	**4.7**	**4.27**	**4.8**
-log10p	1.8	2.3	NaN	2.6	3.5	3.4
Ratio Mt/CP	0.04	0.05	CP only	3.57	1.37	1.05
-log10p	2.7	2.8	2.2	2.1	1.3	0.1
Ratio CP/W	4.5	1.95	CP only	1.31	3.10	4.6
-log10p	3.2	1.7	1.9	0.6	4.4	3.8
Unique peptides	4	30	2	76	35	32
