# Supplementary figures and images for: A Uniquely Complex Mitochondrial Proteome from Euglena gracilis
Source: Mol Biol Evol. 2020 Apr 5;37(8):2173–91. doi: 10.1093/molbev/msaa061 (PMC7403612; doi:10.1093/molbev/msaa061)

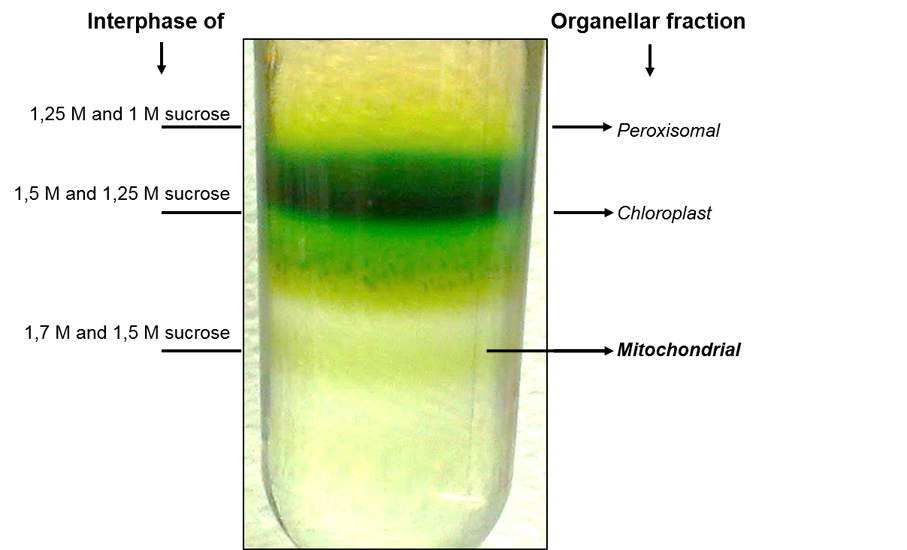

Supplement: msaa061_Supplementary_Data [file msaa061_supplementary_data.zip › msaa061-suppl_data/Suppl. Fig 1..jpg]

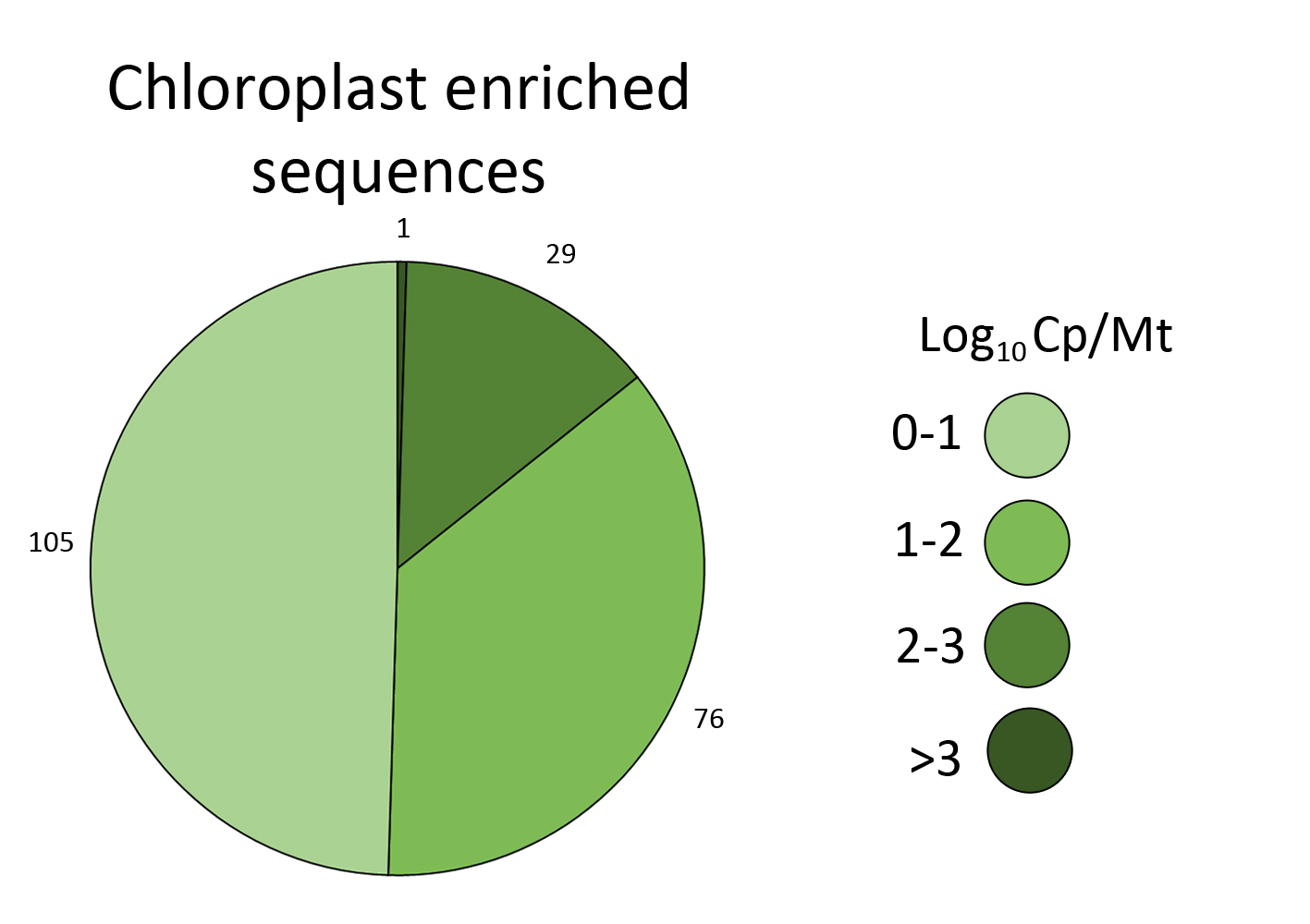

Supplement: msaa061_Supplementary_Data [file msaa061_supplementary_data.zip › msaa061-suppl_data/Suppl. Fig 2.jpg]

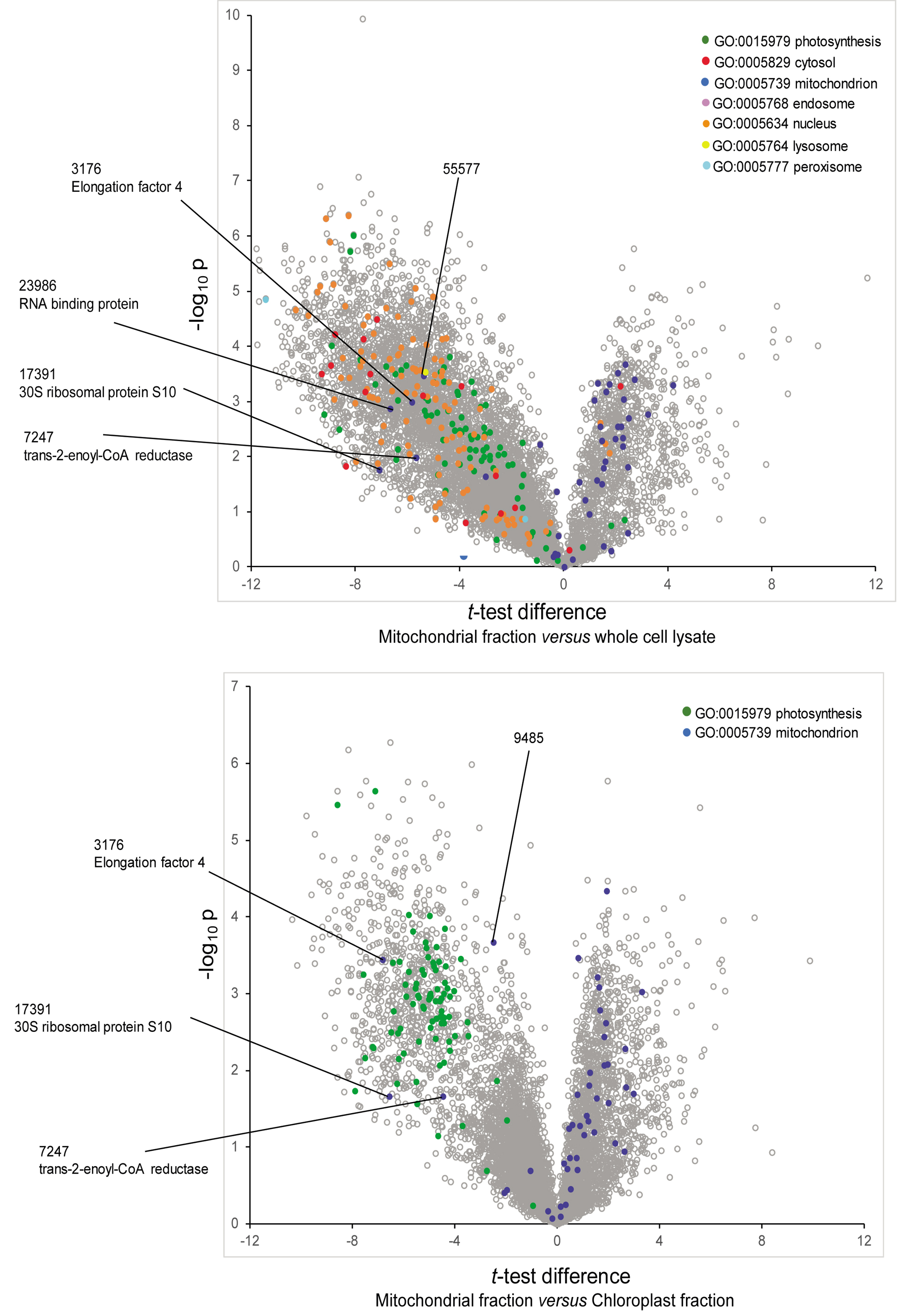

Supplement: msaa061_Supplementary_Data [file msaa061_supplementary_data.zip › msaa061-suppl_data/Suppl. Fig 3.jpg]

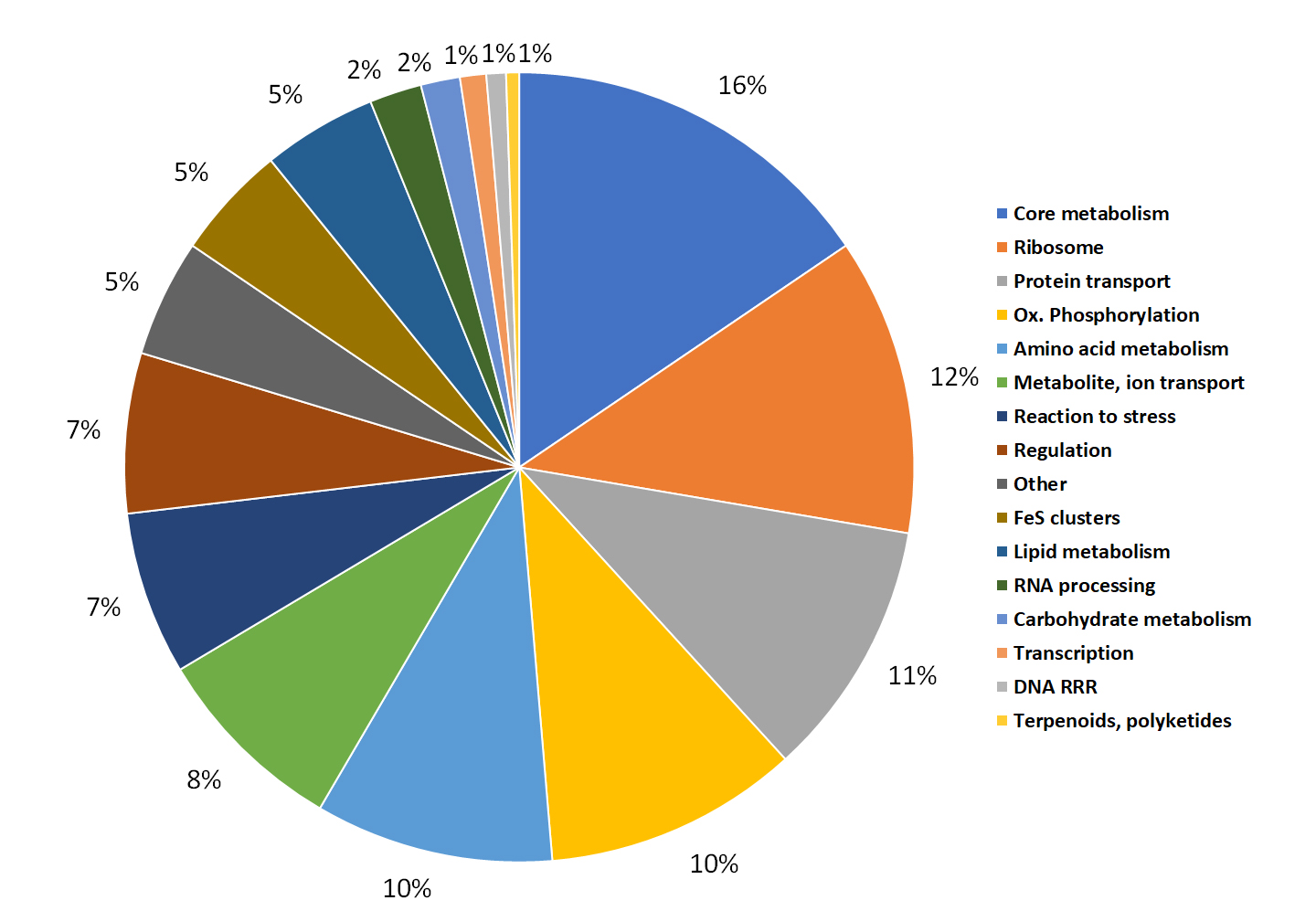

Supplement: msaa061_Supplementary_Data [file msaa061_supplementary_data.zip › msaa061-suppl_data/Suppl. Fig 4.jpg]

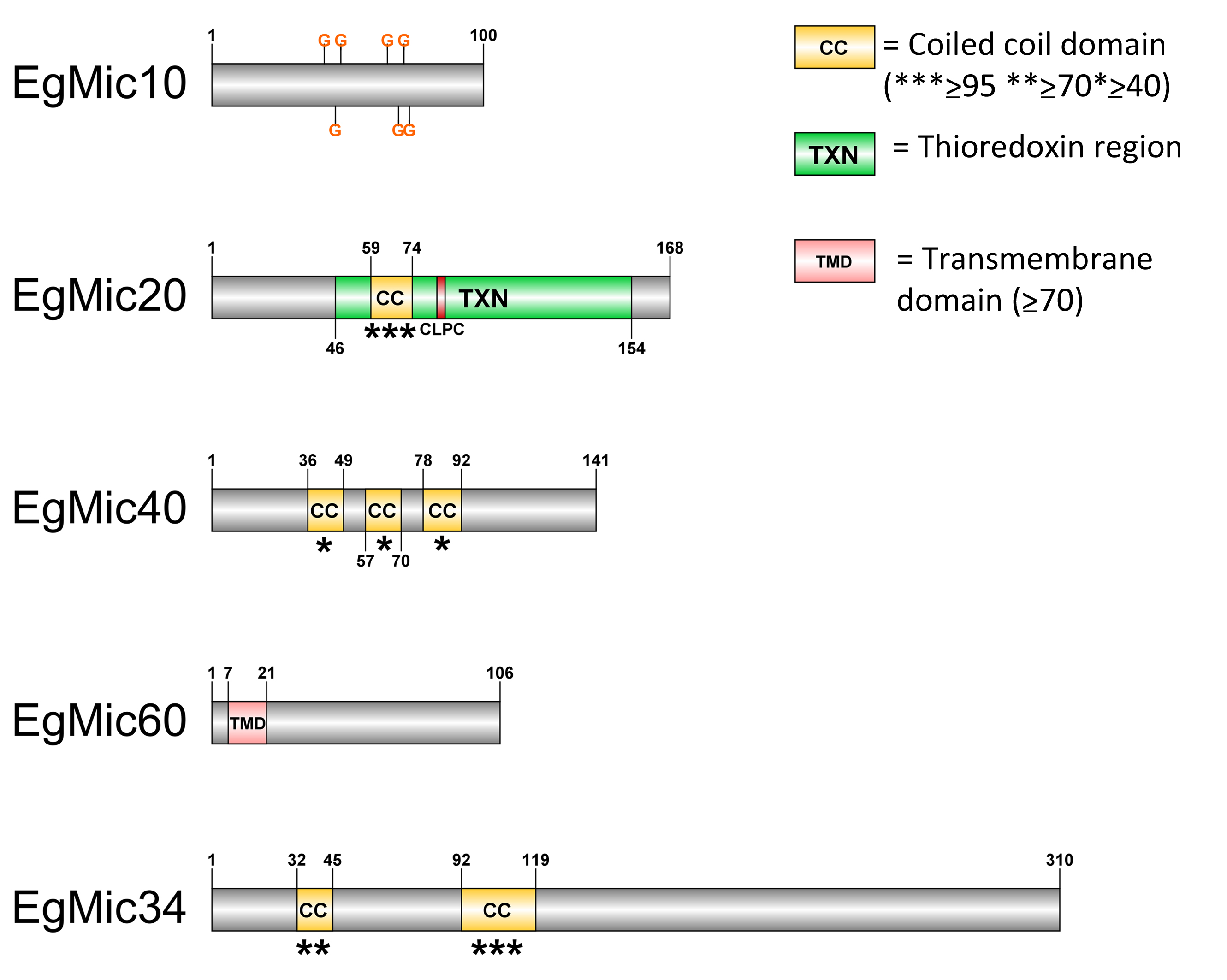

Supplement: msaa061_Supplementary_Data [file msaa061_supplementary_data.zip › msaa061-suppl_data/Suppl. Fig 5..jpg]

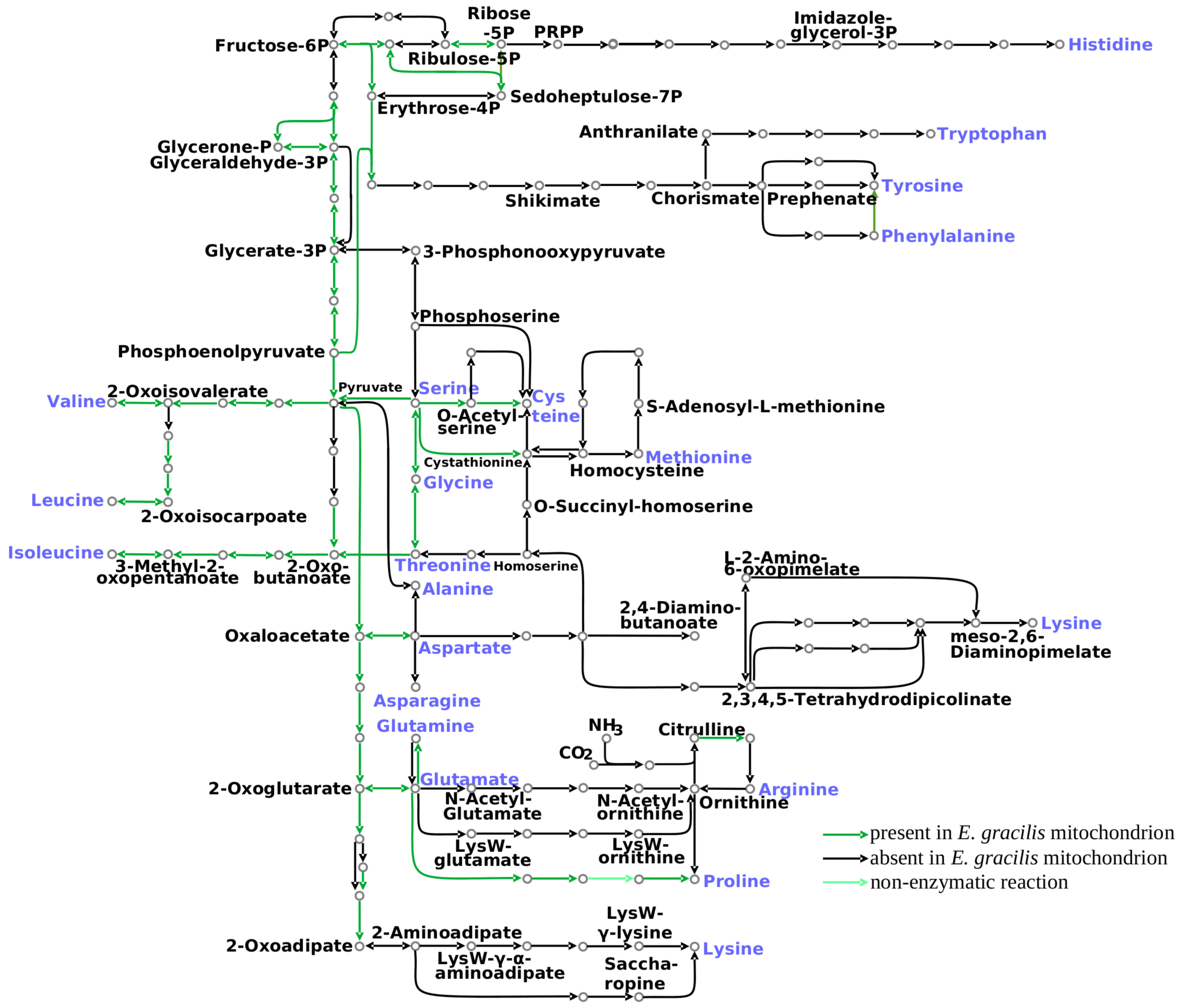

Supplement: msaa061_Supplementary_Data [file msaa061_supplementary_data.zip › msaa061-suppl_data/Suppl. Fig 6..jpg]
